# Low-Lying Excited States of Linear All-*Trans* Polyenes: Insights from Analytic Gradient and Nonadiabatic Coupling Calculations Based on Multireference Configuration Interaction

**DOI:** 10.1021/acs.jctc.6c00075

**Published:** 2026-05-04

**Authors:** Julio C. V. Chagas, Luan G. F. dos Santos, Silmar A. do Monte, Adélia J. A. Aquino, Felix Plasser, Ron Shepard, Péter G. Szalay, Hans Lischka, Francisco B. C. Machado

**Affiliations:** 1 Department of Chemistry, 74360 Aeronautics Institute of Technology, São José dos Campos, SP 12228-900, Brazil; 2 Advanced Scientific Computing and Modeling Laboratory, 74360 Aeronautics Institute of Technology, São José dos Campos, SP 12228-900, Brazil; 3 Department of Chemistry, 3270 Northwestern University, Evanston, Illinois 60208, United States; 4 Department of Chemistry and Biochemistry, 6177 Texas Tech University, Lubbock, Texas 79409, United States; 5 Department of Chemistry, Federal University of Paraíba, João Pessoa, PB 58059-900, Brazil; 6 Department of Mechanical Engineering, 6177 Texas Tech University, Lubbock, Texas 79409, United States; 7 Department of Chemistry, 152008 Loughborough University, Loughborough LE11 3TU, United Kingdom; 8 Chemical Sciences and Engineering Division, 1291 Argonne National Laboratory, Lemont, Illinois 60439, United States; 9 Institute of Chemistry, ELTE Eötvös Loránd University, Budapest H-1117, Hungary

## Abstract

Polyenes serve as a rigorous test for theoretical models and electronic structure methods, playing a key role in advancing computational and theoretical chemistry. Here, we present a high-level theoretical investigation of linear, all-*trans* polyenes using energy gradients and nonadiabatic coupling vectors based on an MR-CISD wave function to describe electronic transitions involving the ground state (1^1^A_g_^–^) and three low-lying excited states (2^1^A_g_^–^, 1^1^B_u_^+^, and 2^1^B_u_^–^) of hexatriene, octatetraene, and decapentaene. This approach enables accurate evaluation of both adiabatic and vertical excitation and emission energies, yielding results in excellent agreement with experiment, as well as locating minima on the crossing seam between adiabatic states. Our results show that vertical excitation energies to the 1^1^B_u_^+^ state are blue-shifted by 0.2–0.3 eV relative to the experimental absorption maximum, whereas the vertical emission energy from the 2^1^A_g_^–^ state is red-shifted by ∼0.2 eV relative to the experimental emission maximum. Upon relaxation from the Franck–Condon geometry, the 2^1^A_g_^–^ state stabilizes by around 1 eV, compared to 0.2–0.3 eV for the 1^1^B_u_^+^ state. An analysis of the S_1_/S_0_ crossing seam in hexatriene shows that its minimum involves asymmetric backbone deformations and provides an efficient channel for ultrafast internal conversion to the ground state, consistent with the absence of detectable fluorescence in this molecule. These results demonstrate the power of analytic gradients and nonadiabatic coupling vectors based on an MR-CISD wave function for accurately characterizing the electronic structure and photophysics of polyenes.

## Graphical abstract

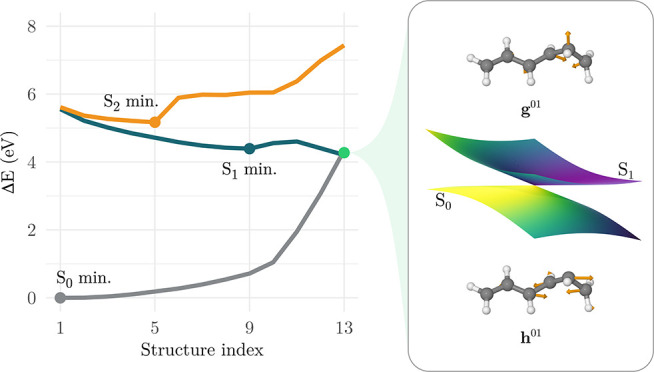

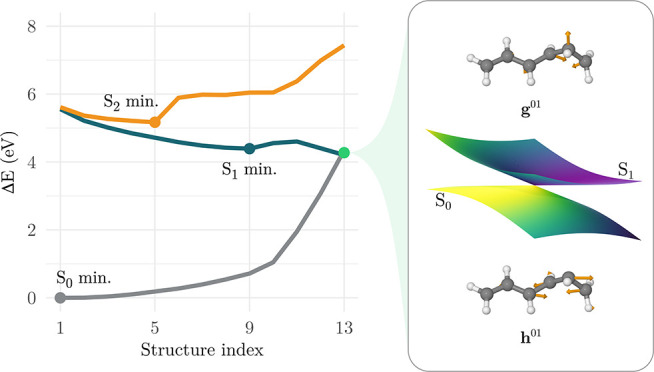

## 1 Introduction

The investigation of linear all-*trans* polyenes has long been a cornerstone of molecular photophysics due to their unique spectral properties, which stem from the intricate nature of their low-lying excited states. Among the manifold of excited states, the two lowest-lying singlet states-the covalent 2^1^A_g_^–^ state, and the ionic 1^1^B_u_^+^ state-are focal points for experimental^1–11^ and computational studies.^12–28^ Part of the great interest in polyenes is related to the fact that, beyond their chemical and biological relevance^29,30^ polyenes provide a rigorous testing ground for theoretical frameworks and electronic structure methodologies.

The focus of this work is on *trans,trans*-1,3,5-hexatriene, all-*trans*-1,3,5,7-octatetraene, and all-*trans*-1,3,5,7,9-decapentaene−hereafter referred to as hexatriene, octatetraene, and decapentaene. The 1^1^B_u_^+^ ← 1^1^A_g_^–^ transition is dipole-allowed, which enables its detection in single-photon absorption experiments. The origin peak−which is also the strongest peak, has been unambiguously observed in the gas phase for hexatriene,^1,8^ octatetraene,^3,5,7^ and decapentaene.^4^ On the other hand, the 2^1^A_g_^–^ state is dipole forbidden, requiring more sophisticated detection techniques, such as two-photon absorption experiments in free jet expansions.^11^ Nevertheless, it has been demonstrated that in octatetraene, the one-photon cross section is sufficiently large to allow the detection and study of the 2^1^A_g_^–^ state.^11^

Ionic states, like the 1^1^B_u_^+^, are typically composed of singly excited configurations, whereas covalent states, like the 2^1^A_g_^–^, are substantially composed of doubly excited configurations.^18,19,28,29^ The different characters of these states create theoretical challenges in uniformly describing the electronic spectra of these molecules: single-reference methods like time-dependent density functional theory (TD-DFT),^17,21,30^ algebraic diagrammatic construction of second order (ADC(2)),^20^ and equation of motion coupled-cluster (EOM-CCSD)^25^ adequately describe ionic states but have difficulties to capture nondynamic correlation effects present in covalent states. The multiconfiguration self-consistent field (MCSCF) method provides accurate results for covalent states but exhibits systematic limitations when applied to ionic states.^28,31,32^ Indeed, a reasonable description of ionic states at the multireference (MR) level requires an extensive treatment of the σ-π electron correlation.^28,33^

In this context, perturbative treatments, such as complete active space second-order perturbation theory (CASPT2)^12,13,16,23,27^ and N-electron valence perturbation theory (NEVPT2),^23,27^ as well as variational methods like multireference configuration interaction with singles and doubles (MR-CISD),^18,28^ MR-CISD+P (including Pople’s size-extensivity correction correction)^34^ and multireference averaged quadratic coupled-cluster (MR-AQCC),^18,28^ are good alternatives for obtaining a balanced description of the two lowest-lying excited states of polyenes with differing characters. As a further alternative, we have recently developed a scaled CASSCF approach intended to mimic σ–π electron correlation by screening the exchange integrals involved.^35^ In recent work, uncontracted MR-CISD, MR-CISD+P, and MR-AQCC methods have been employed to accurately compute the vertical excitation energies of all-trans polyenes, from hexatriene up to dodecahexaene.^28^ To further describe their electronic spectra and gain deeper insight into their spectroscopic behavior, it is necessary to locate the minima of the excited-state potential energy surfaces. This enables the calculation of adiabatic excitation energies as well as vertical emission energies from the excited-state minima, which is one of the aims of this work.

Recent methodological developments have focused on obtaining analytic energy gradients and nonadiabatic coupling vectors at correlated multireference levels of theory.^36–39^ In this context, the multiconfiguration pair-density functional theory (MC-PDFT) method^39^ has emerged as a cost-effective approach. Owing to the lack of analytic energy gradients in many MR methods, excited-state geometry optimizations and excited-state dynamics studies are often performed at the CASSCF level, followed by single-point calculations at a higher level of theory. This approach has been widely used. Nakayama et al.,^16^ studied low-lying excited states of polyenes ranging from ethylene to octatetraene using CASPT2 based on CASSCF-optimized geometries. Serrano-Andrés and coauthors,^12^ as well as Angeli and Pastore,^23^ using the same approach, studied octatetraene. Recently, Guareschi and Angeli^27^ successfully obtained the optimized geometries of the ground state and excited 2^1^A_g_^–^ state of hexatriene via numerical gradients based on a NEVPT2 calculation. An important finding from these studies is that upon geometry relaxation, the covalent 2^1^A_g_^–^ state, which lies very close to the ionic state at the Franck–Condon geometry, is highly stabilized.

In addition to describing the electronic spectra of these polyenes by calculating adiabatic and vertical absorption and emission energies, this work also investigates the ultrafast photochemical processes that take place in hexatriene upon excitation to the bright 1^1^B_u_^+^ state, focusing on the conical intersections governing the ultrafast decay to the ground state. Unlike the larger members of this series, which exhibit significant fluorescence yields, with excited-state lifetimes on the nanosecond scale, hexatriene represents a distinct case. Photoexcitation from the ground state to the bright 1^1^B_u_^+^ state in hexatriene initiates an ultrafast radiationless process that begins in the 1^1^B_u_^+^ state, which has a lifetime of ∼40 fs.^40,41^ The system then evolves via internal conversion to the dark 2^1^A_g_^–^ state, with a lifetime of ∼270 fs,^42^ and finally undergoes internal conversion back to the ground state. This ultrafast dynamics arises from the presence of potential-energy surface crossings that connect 1^1^B_u_^+^ to 2^1^A_g_^–^, and 2^1^A_g_^–^ to 1^1^A_g_^–^.^15^

The variational character of the uncontracted MR-CISD method, as implemented in COLUMBUS,^43–46^ enables efficient evaluation of analytic energy gradients and nonadiabatic coupling vectors for any selection of electronic states.^47–49^ The computational cost of gradient evaluation is only a small fraction of that required for a single-point energy calculation. In this work, the minima of the ground state (1^1^A_g_^–^) and the excited states (2^1^A_g_^–^, 1^1^B_u_^+^ and 2^1^B_u_^–^) were calculated for hexatriene, octatetraene, and decapentaene using analytic energy gradients based on the MR-CISD wave function, enabling a consistent and uniform description of adiabatic and vertical electronic transition energies in these compounds. By computing analytic nonadiabatic coupling vectors, we also located and characterized the minimum of the crossing seam (MXS) between S_1_ and S_0_ in hexatriene, thereby identifying the conical intersection responsible for ultrafast photochemical processes in this molecule at a high level of theory.

## 2 Computational Details

The equilibrium geometries of the ground state (1^1^A_g_^–^) and the singlet excited states 2^1^A_g_^–^, 1^1^B_u_^+^, and 2^1^B_u_^–^ of hexatriene, octatetraene, and decapentaene were obtained via analytic energy gradients based on the MR-CISD wave function. While energy gradients and nonadiabatic coupling vectors are calculated at the MR-CISD level, the Pople (+P) energy correction^34^ was applied to all results presented in this work to account for size-extensivity errors inherent to MR-CISD. Except for the MXS between S_0_ and S_1_ in hexatriene reported in Section 3.4, where the *C*_1_ point group was used, all geometry optimizations were performed under *C*_2h_ symmetry. Cartesian coordinates for all optimized structures are given in the Supporting Information (SI).

At the CASSCF level, equal-weight state averaging was performed over six electronic states: 1^1^A_g_^–^, 2^1^A_g_^–^, 1^1^B_u_^+^, 2^1^B_u_^–^, 1^3^A_g_^–^, and 1^3^B_u_^–^, following our previous work on vertical excitations.^28^ This state-averaging scheme was employed in all calculations, with the exception of the MXS optimizations, for which only S_0_ and S_1_ were included. The CASSCF computations employed a standard valence active space encompassing all valence π-electrons and π-orbitals of each molecule. The molecular orbitals obtained from this state-averaging procedure were subsequently used in the MR-CISD calculations. The reference space at the MR-CISD level was constructed by partitioning the π-valence space into three subsets: a CAS­(6,6), a complementary restricted active space (RAS) for the remaining strongly occupied π orbitals, and a corresponding auxiliary (AUX) space for weakly occupied orbitals (Table 1). For more details see ref 28. Single excitations were allowed from the RAS into the CAS and AUX spaces, as well as from the CAS into AUX orbitals. The specific schemes adopted in our calculations are listed in Table 1. Based on this reference space, single and double excitations into all virtual orbitals were generated, forming the expansion space of the MR-CISD wave function. In all calculations, only the core orbitals were frozen. Generalized interacting space restrictions^50^ were applied at the MR-CISD level.

**Table 1:** Active Reference Space Scheme Utilized at the MR-CISD Level for Polyenes with *N* π-Electrons

*N*	active space scheme
6	MR-CISD(CAS(6,6))
8	MR-CISD(RAS(1)CAS(6,6)AUX(1)
10	MR-CISD(RAS(2)CAS(6,6)AUX(2))

Excited-state geometry optimizations were performed at the MR-CISD level using analytic gradients developed in refs.^47^ and^51^. MXS optimizations^52^ were carried out at the MR-CISD level using analytic nonadiabatic coupling vectors^53^ and the direct inversion in the iterative subspace (GDIIS)^54–56^ approach.

To obtain an instructive presentation of the photochemical pathway in hexatriene, geodesic interpolations were performed between the following pairs of reference geometries: the ground-state minimum (S_0_ minimum) to the minimum on the S_2_ potential energy surface (S_2_ minimum), and from the S_2_ minimum to the minimum on the S_1_ surface (S_1_ minimum). From the S_1_ minimum, distinct pathways were constructed independently, connecting the S_1_ minimum to the two MXS discussed in this work. For each adjacent pair of reference structures, a separate geodesic interpolation was carried out using five structures, where the first and last structures correspond to the optimized end point geometries and the three intermediate structures were generated by the geodesic interpolation algorithm. The geodesic interpolation was performed following the method by Zhu, Thompson, and Martínez.^57^ In this approach, the interpolation between two molecular structures is formulated as a geodesic problem on a Riemannian manifold defined by redundant internal coordinates. By explicitly accounting for the internal coordinate metric, this method yields smooth and physically meaningful structural changes between end point geometries and avoids artifacts associated with linear Cartesian interpolation.

Basis set effects were also investigated. Based on the optimized geometries of the ground and excited states obtained with the double-ζ cc-pVDZ basis set, single-point calculations using the triple-ζ cc-pVTZ basis set^58^ were carried out. Extrapolation to the complete basis set (CBS) limit was performed using the two-point fit approach^59^ (eq 1) for energy splittings obtained at the MR-CISD+P level.

$$E_{\mathrm{XY}}^{\infty }=\frac{E_{X}X^{3}-E_{Y}Y^{3}}{X^{3}-Y^{3}}$$ 1

In eq 1, E_XY_^∞^ represents the CBS limit excitation energy, *X* and Y are the cardinal numbers for the respective basis sets, and E_X_ and E_Y_ denote the excitation energies obtained for those individual basis sets. The cc-pVTZ basis set without the *f*-function, i.e., with contraction (10*s*,5*p*,2*d*)/[4*s*,3*p*,2*d*] (cc-pVTZ’), was used on carbon atoms in these single-point calculations. The cc-pVDZ basis set was used on hydrogen atoms in all cases.

All calculations were performed using the described formalism implemented in the COLUMBUS program system,^43–46^ with integrals calculated by the Dalton program.^60^

## 3 Results and Discussion

### 3.1 Adiabatic Excitation Energies

We begin our discussion by presenting results for adiabatic excitation energies, calculated as the difference between the ground state and excited state minima. From the experimental point of view, this is comparable to the energy of the 0–0 excitation band, although only the electronic contribution is considered here. The inclusion of zero-point vibrational energy has been studied in hexatriene^27^ and octatetraene,^23^ and it has been demonstrated that vibrational corrections typically reduce excitation energies by 0.1–0.2 eV. The adiabatic excitation energies of the singlet excited states of hexatriene, octatetraene, and decapentaene are presented in Table 2.

**Table 2:** Adiabatic Excitation Energies (eV) of Singlet States in Polyenes with *N* π-Electrons, Computed at the MR-CISD+P Level with the cc-pVDZ Basis Set and Geometries Optimized at the MR-CISD Level^o^

*N*	State	MR-CISD+P	CASPT2	NEVPT2	Exp. 0–0
6	2^1^A_g_^–^	4.365 (4.471)	4.195^a^, 4.17^b^	4.341, 4.347^g^	≤4.22^j^
	1^1^B_u_^+^	5.335 (5.199)	5.177^c^, 4.84^b^	5.265, 5.165^h^	4.93^k^
	2^1^B_u_^–^	5.747 (5.861)			
8	2^1^A_g_^–^	3.645 (3.748)	3.44^d^, 3.50^e^	3.77, 3.75^i^	3.59^l^
	1^1^B_u_^+^	4.698 (4.787)	4.33^d^, 4.34^e^	4.56, 4.44^i^	4.41^m^
	2^1^B_u_^–^	5.038 (5.168)	5.39^f^	5.41, 5.36^i^	
10	2^1^A_g_^–^	3.122 (3.222)	2.99^e^		3.10^n^
	1^1^B_u_^+^	4.236 (4.192)	3.88^e^		4.02^n^
	2^1^B_u_^–^	4.389 (4.522)			

a Geometry optimized at the NEVPT2/CAS­(6,6) level; single-point energy at the CASPT2/CAS­(6,14) level.^27^

b Geometry optimized at the CASSCF/CAS­(6,6) level; single-point energy at the CASPT2/CAS­(6,12) level.^16^

c Geometry optimized at the RASSCF/RAS(12)­CAS­(6,6)­AUX(12) level; single-point energy at the CASPT2/CAS­(6,14) level.^27^

d Geometry optimized at the CASSCF/CAS­(8,8) level; single-point energy at the CASPT2/CAS­(8,16) level.^23^

e Geometry optimized at the CASSCF/CAS­(*N*,*N*) level; single-point energy at the CASPT2/CAS­(*N*,*N*) level, where *N* is the number of carbon atoms.^16^

f Geometry optimized at the CASSCF/CAS­(8,8) level; single-point energy at the CASPT2/CAS­(8,12) level.^23^

g Geometry optimized at the NEVPT2/CAS­(6,6) level; single-point energy at the SC- and PC-NEVPT2/CAS­(6,14) levels, respectively.^27^

h Geometry optimized at the RASSCF/RAS(12)­CAS­(6,6)­AUX(12) level; single-point energy at the SC- and PC-NEVPT2/CAS­(6,14) levels, respectively.^27^

i Geometry optimized at the CASSCF/CAS­(8,8) level; single-point energy at the SC- and PC-NEVPT2/CAS­(8,16) levels, respectively.^23^

j Estimated origin of absorption of the multiphoton ionization spectrum.^9^

k Origin of absorption.^1,8^

l Origin of absorption.^3,11^

m Origin of absorption.^3,5,7^

n Origin of absorption.^4^

o Extrapolated values to the complete basis set limit (Δ*E*_*DT*_^∞^) are given in parentheses. Available theoretical reference values and experimental data are provided for comparison.

The experimentally observed origin of the bright state of hexatriene in the gas phase is located at 4.93 eV.^1,8^ The adiabatic excitation energy computed at the MR-CISD+P level is 5.335 eV, which is 0.41 eV higher than the experimental 0–0 excitation band. At the CBS limit, this value is reduced by 0.14 eV, and the estimated excitation energy is 5.199 eV. The MR-CISD+P results are in line with both NEVPT2 and CASPT2 data (Table 2). Experimentally, the origin of the 2^1^A_g_^–^ state in hexatriene has not been unambiguously determined for the *trans* isomer. Buma and coauthors^9^ estimated the origin of the 2^1^A_g_^–^ state to have an energy ≤ 4.22 eV. Corroborating this result, it is generally assumed^61^ that the origin of this state lies within a few hundred wavenumbers of the corresponding state in the *cis* isomer of hexatriene, which is unambiguously found at 4.26 eV.^9,62^ The MR-CISD+P results provide an excitation energy of 4.365 eV, strong evidence of the region where this state lies, in quite good agreement with the estimated origin. Supporting these results, the NEVPT2 values are within 0.02 eV of the MR-CISD+P results. CASPT2 calculations, on the other hand, give slightly lower excitation energies, with values of 4.20 and 4.17 eV for this transition (Table 2).

As for hexatriene, the origin of the 1^1^B_u_^+^ ← 1^1^A_g_^–^ transition in octatetraene is observed in gas-phase one-photon absorption experiments.^3,5,7^ Recent experiments yield a value of 4.41 eV.^5,7^ Our calculated adiabatic excitation energy is 4.698 eV, a value 0.29 eV higher than the absorption origin. The origin of the 2^1^A_g_^–^ ← 1^1^A_g_^–^ transition has been precisely observed in the gas phase by means of two-photon absorption experiments.^11^ Additionally, the origin of this transition has been measured in the condensed phase and extrapolated to the gas phase.^3^ Both experimental results suggest that the minimum of the 2^1^A_g_^–^ state lies 3.59 eV above the minimum of the ground state. Our results yield 3.645 eV−in remarkable good agreement with experiment. Overall, the MR-CISD+P results show good consistency with previous CASPT2 and NEVPT2 calculations (Table 2).

Experimental and theoretical data for decapentaene are less abundant. The origin of the 1^1^B_u_^+^ ← 1^1^A_g_^–^ transition is directly observable in the gas phase and is also shown to coincide with the absorption maximum of the spectrum at 4.02 eV.^4^ The adiabatic excitation energy calculated at the MR-CISD+P level lies 0.22 eV above this, at 4.236 eV, showing good accuracy. The 2^1^A_g_^–^ ← 1^1^A_g_^–^ transition energy has only been measured in the condensed phase and extrapolated to the gas phase.^4^ This approach applied to the ionic state−both in octatetraene^3^ and decapentaene^4^−is shown to be reliable, yielding results in good agreement with gas-phase data. The accepted extrapolated experimental value is 3.10 eV. The adiabatic excitation energy calculated at the MR-CISD+P level is 3.122 eV, in excellent agreement with the experimental data. CASPT2 yields a similarly good description, with a deviation of 0.11 eV (Table 2).

In summary, the MR-CISD+P adiabatic excitation energies (Table 2) agree closely with the experimentally measured origin band and other theoretical results. The MR-CISD+P results closely align with previous NEVPT2 predictions, whereas CASPT2 tends to give slightly lower transition energies. Across the systems, better agreement with experiment is observed for the 2^1^A_g_^–^ ← 1^1^A_g_^–^ transitions than for the 1^1^B_u_^+^ ← 1^1^A_g_^–^ transitions. This stems from the high demands placed by the ionic state in terms of σ-π electron correlation and high-order excitations needed to accurately describe the 1^1^B_u_^+^ state.^28,32^ Performing single-point calculations using the triple-ζ basis set cc-pVTZ’ on the relaxed geometries obtained with the double-ζ basis set−except for hexatriene, where slightly better agreement with experiment is observed at the CBS limit−has little impact on adiabatic excitation energies (Table S1).

### 3.2 Vertical Excitation and Emission Energies

In this section, we present and discuss the vertical excitations to the bright 1^1^B_u_^+^ state, as well as the vertical emission energies from the two lowest singlet excited states of the polyenes (1^1^B_u_^+^ and 2^1^A_g_^–^), estimating the fluorescence intensity maximum based on a vertical transition from the excited-state minimum to the ground state. Vertical excitation energies were obtained by calculating the energies of the excited states at the equilibrium geometry of the ground state. These values are generally compared to the absorption band maximum (λ_abs_^max^) under the assumption of classical vibrations.^63^ For the systems studied, λ_abs_^max^ also corresponds to the origin band.^1,4,6^ Vertical excitation energies are presented in Table S2. Conversely, vertical emission energies were evaluated at the minima of the corresponding excited states. An experimental estimate of the vertical emission energy is the wavelength corresponding to the maximum of the emission spectrum (λ_emi_^max^). In Table 3, the absorption and emission energies, calculated at the MR-CISD+P level, are presented.

**Table 3:** Absorption (Δ*E*_abs_) and Emission (ΔE_emi_) Energies (eV) for Polyenes with *N π*-Electrons Computed at the MR-CISD+P Level Using the cc-pVDZ Basis Set^h^

	MR-CISD+P			Exp.		
*N*	Δ*E*_abs_ ^a^	Δ*E*_emi_	Δ*E*_emi_	Δ*E*_abs_	Δ*E*_emi_	Δ*E*_emi_
	1^1^B_u_^+^ ← 1^1^A_g_^–^	1^1^A_g_^–^ ← 2^1^A_g_^–^	1^1^A_g_^–^ ← 1^1^B_u_^+^	1^1^B_u_^+^ ← 1^1^A_g_^–^	1^1^A_g_^–^ ← 2^1^A_g_^–^	1^1^A_g_^–^ ← 1^1^B_u_^+^
6	5.442	3.630	5.140	4.93^b^		
8	4.825	2.963	4.522	4.41^c^	3.1^d^	4.41^e^
10	4.364	2.500	4.072	4.02^f^	2.7^g^	

a Values computed at the basis set limit. See Table S2.

b Origin of absorption for the isolated compound.^1^

c Origin of absorption for the isolated compound.^6^

d Vibronic patterns assumed similar in gas and condensed phases.^4^

e Emission band maximum for the isolated compound.^6^

f Origin of absorption for the isolated compound.^4^

g Vibronic patterns assumed similar in gas and condensed phases.^4^

h Geometries were optimized at the MR-CISD level for the ground state (absorption) and the respective emitting state (emission). Experimental values for the absorption (λ_abs_^max^) and emission (λ_emi_^max^) band maxima are provided for comparison.

For vertical excitation, we use the MR-CISD+P result extrapolated to the CBS limit, as the basis set can significantly influence the 1^1^B_u_^+^ ← 1^1^A_g_^–^ vertical excitation energy due to the sensitivity of the 1^1^B_u_^+^ state to electron correlation and orbital relaxation effects.^28,32^ The results obtained for each basis set are presented in Table S2. For the studied compounds, our results predict vertical excitation energies higher than the absorption band maxima, exceeding the experimental estimates by 0.51 eV in hexatriene, 0.42 eV in octatetraene, and 0.34 eV in decapentaene. The larger deviation observed for the smaller systems is attributed to the greater role of σ-π electron correlation and the higher-order excitations needed to properly describe the exchange interaction arising from the HOMO → LUMO excitation in systems with fewer electrons.^64^ Previous studies have shown that vertical excitation energies from the ground-state minimum are typically blue-shifted relative to the absorption band maximum by approximately 0.2 eV^63,65^ As will be discussed in the next section, this is consistent with our findings. Taking this into account, the MR-CISD+P values show good agreement with the experimental estimates. Similar results (Table S2) were obtained by Nakayama and co-workers^16^ and by Angeli et al.^23,27^ at the CASPT2 and NEVPT2 levels of theory.

For hexatriene, which does not exhibit detectable fluorescence, the MR-CISD+P results predict a vertical emission energy of 3.63 eV, corresponding to 342 nm. Octatetraene is the smallest unsubstituted polyene with detectable fluorescence. It is worth noting that this molecule exhibit gas-phase dual fluorescence.^10,66,67^ The strongest luminescence band is structured and occurs with an anomalously small Stokes shift (ca. 20 nm), which supports a 1^1^A_g_^–^ ← 1^1^B_u_^+^ assignment for the fluorescence.^3,6^ At the MR-CISD+P level, the predicted Stokes shift is 17.2 nm, in excellent agreement with experiment. The 1^1^A_g_^–^ ← 2^1^A_g_^–^ transition displays a broad and unstructured band and has a much lower intensity, approximately 6% of the maximum intensity of the strongest band.^10^ For this transition, our MR-CISD+P results indicate that the vertical emission energy is red-shifted relative to the maximum of the emission spectrum; specifically, the calculated vertical emission energy is 0.14 eV lower than the experimental emission band maximum. Similarly, for decapentaene, the vertical 1^1^A_g_^–^ ← 2^1^A_g_^–^ emission energy is 0.20 eV lower than the experimental emission band maximum.

### 3.3 Relaxation Energies and Equilibrium Geometries

From the analysis of both adiabatic (Table 2) and vertical excitation energies (Table 3, Table S2), the most important observation is the substantial stabilization of the covalent 2^1^A_g_^–^ state relative to the ground-state minimum upon relaxation to its equilibrium geometry. By contrast, the ionic 1^1^B_u_^+^ state undergoes a much less pronounced stabilization. Figure 1 illustrates the stabilization energies for the two states.

**Figure 1:**
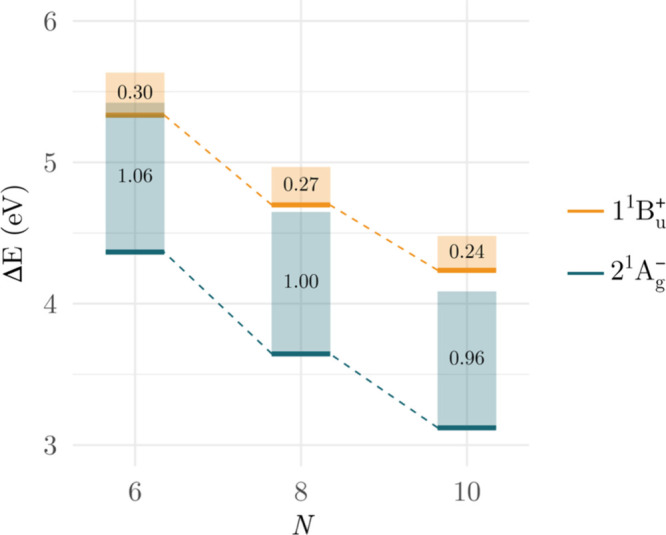
Relaxation energies (eV) in polyenes with *N* π-electrons computed at the MR-CISD+P level using the cc-pVDZ basis set. Geometry optimizations were performed at the MR-CISD level. Relaxation energies*—*represented by the bar sizes and numeric values*—* are defined as the energy differences between vertical excitation energies (top of the bars) and adiabatic excitation energies (bottom of the bars). Numerical data from Table S3.

The relaxation energy observed for the covalent 2^1^A_g_^–^ state ranges from 1.06 eV for hexatriene to 0.96 eV for decapentaene (Figure 1). This substantial relaxation energy reflects the significant difference in nuclear equilibrium geometries of the states involved. The geometry of the 2^1^A_g_^–^ state exhibits a bond-length alternation opposite that of the ground state (Figure 2, Table S4). Specifically, while the ground state features a terminal C–C double bond (Figure 2a), the 2^1^A_g_^–^ state instead shows a characteristic terminal C–C single bond (Figure 2b).

**Figure 2:**
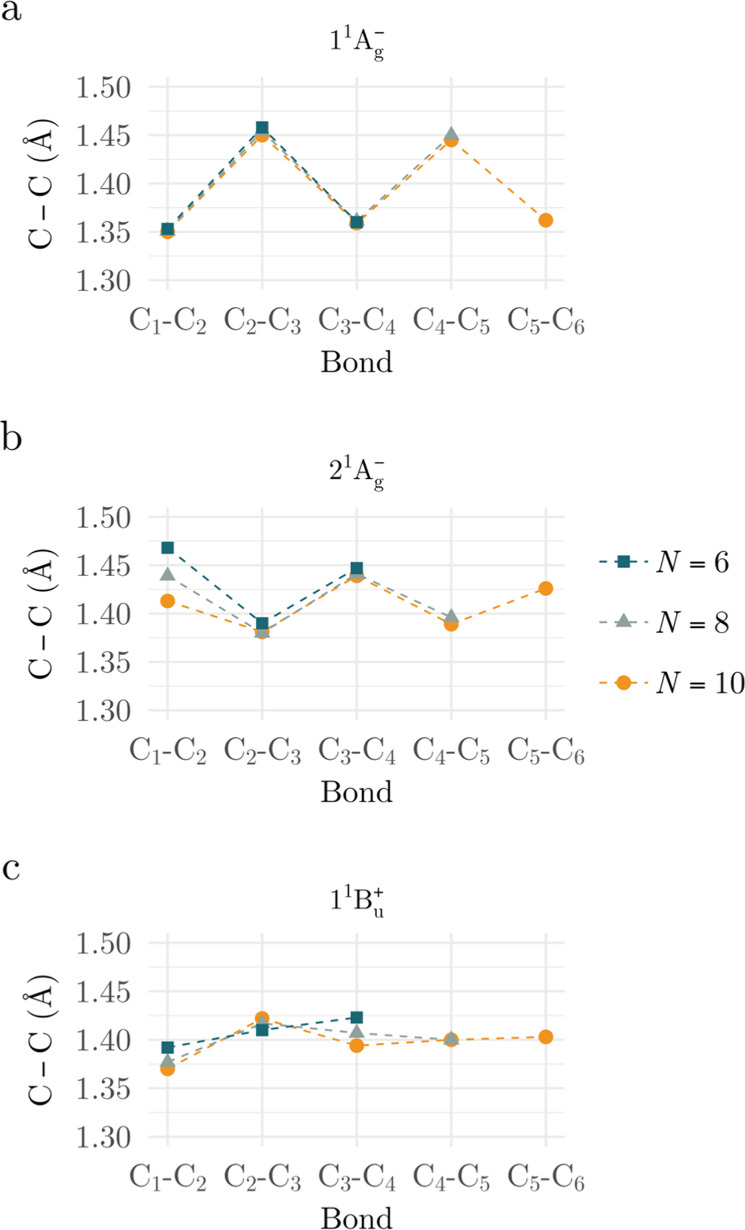
C–C bond lengths of the optimized geometries obtained via analytic energy gradients at the MR-CISD/cc-pVDZ level for the states: (a) 1^1^A_g_^–^, (b) 2^1^A_g_^–^, and (c) 1^1^B_u_^+^ of polyenes with *N* π-electrons. Numerical data from Table S3.

In contrast, the ionic 1^1^B_u_^+^ state is only slightly stabilized at its minimum relative to its energy at the Franck–Condon geometry. The relaxation energy for this state ranges from 0.30 eV for hexatriene to 0.24 eV for decapentaene (Figure 1). Unlike the covalent state, which shows a distinct single–double bond alternation, the ionic state exhibits a more uniform bond-length distribution (Figure 2c, Table S4).

The geometries obtained at the CASSCF level by Nakayama et al.,^16^ agree well with our MR-CISD results (Table S4). Across all three states of the studied compounds, no differences larger than 0.051 Å in C–C bond lengths were found between the respective geometries at the two levels. The ground state and the 2^1^A_g_^–^ excited state of hexatriene have also been previously optimized using the high-level multireference NEVPT2 method with numerical gradients.^27^ The resulting bond distances are in good agreement with those obtained from MR-CISD calculations.

The results obtained here show that the vertical excitation into the bright 1^1^B_u_^+^ state is followed by a structural relaxation of approximately 0.2–0.3 eV (Figure 1). Experimentally, for these systems, the 1^1^B_u_^+^ ← 1^1^A_g_^–^ absorption band maximum is also known to correspond to the 0–0 origin band.^3,4,8^ Although vertical excitation energies are often compared directly with this band maximum, such comparisons implicitly assume negligible vibrational effects.^63^ The observed relaxation energies suggest that vertical excitation energies from the ground-state equilibrium geometry are systematically blue-shifted by 0.2–0.3 eV relative to the absorption band maximum. This shift has been observed and previously reported in high-level theoretical studies.^63,65^

The 2^1^B_u_^–^ state, similarly to the covalent 2^1^A_g_^–^ state, undergoes significant geometric relaxation, with relaxation energies ranging from 0.85 eV for hexatriene to 0.78 eV for decapentaene (Table S3, Figure S2). The optimized geometries of this state exhibit a nearly uniform bond-length distribution at the terminal C–C bonds, while the central C–C bonds display a bond-order pattern opposite to that of the ground state (Table S4 and Figure S4).

### 3.4 A Closer Look at Hexatriene

Figure 3 shows the ground- and excited-state energies computed at the optimized ground, 1^1^B_u_^+^, and 2^1^A_g_^–^ minima, along with the relative energies of the MXS structures. The figure reveals a significant stabilization of the 2^1^A_g_^–^ state along this sequence of structures. The corresponding transition densities with respect to the ground state are also displayed. They show that the characteristic in-plane polarization rings derived from the σ-orbital contributions for the ionic state^28,31,68^ also appear in the transition densities computed at the excited-state minima.

**Figure 3:**
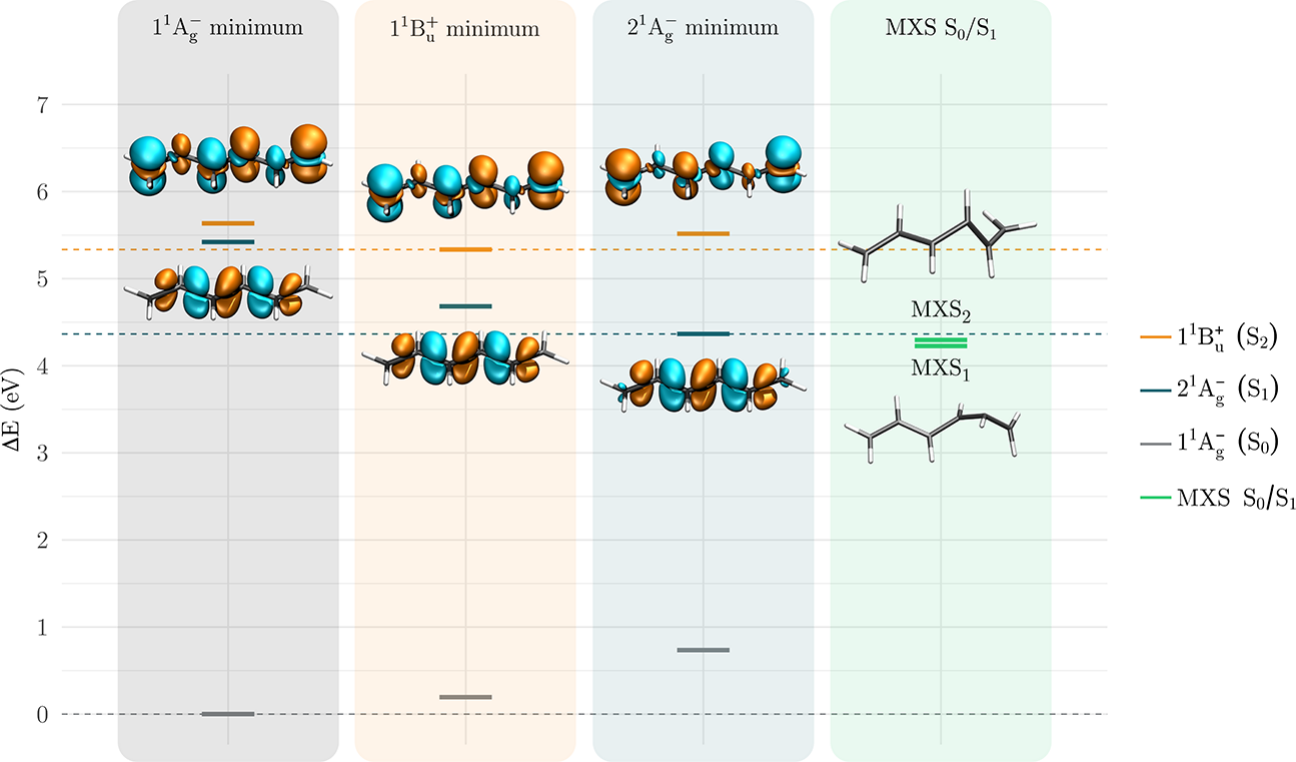
Relative energies of the 1^1^A_g_^–^ (S_0_), 2^1^A_g_^–^ (S_1_), and 1^1^B_u_^+^ (S_2_) states of hexatriene. The two minima of the S_0_/S_1_ crossing seam (MXS) are also indicated. All values were computed at the MR-CISD+P/SA6-CAS­(6,6)/cc-pVDZ level and are referenced to the 1^1^A_g_^–^ minimum. Transition densities between the ground state and each excited state are shown at their respective optimized geometries.

Based on MR-CISD analytic gradients and nonadiabatic coupling vectors, we located and characterized two different MXS structures (MXS_1_ and MXS_2_; Figure 4) for the photodeactivation of hexatriene from S_1_ to S_0_. Both MXS structures involve strong distortions from planarity, whereas the initial decay path from S_2_ to S_1_ and the subsequent relaxation along the S_1_ potential energy surface proceed essentially through symmetric deformations of the molecular backbone.^15^ MXS_1_ shows a distortion of the terminal CH_2_ group and is about 0.14 eV lower in energy than the S_1_ minimum. By choosing a different starting geometry taken from ref. 15, we could locate MXS_2_ between S_1_ and S_0_, about 0.07 eV lower in energy than the S_1_ minimum.

**Figure 4:**
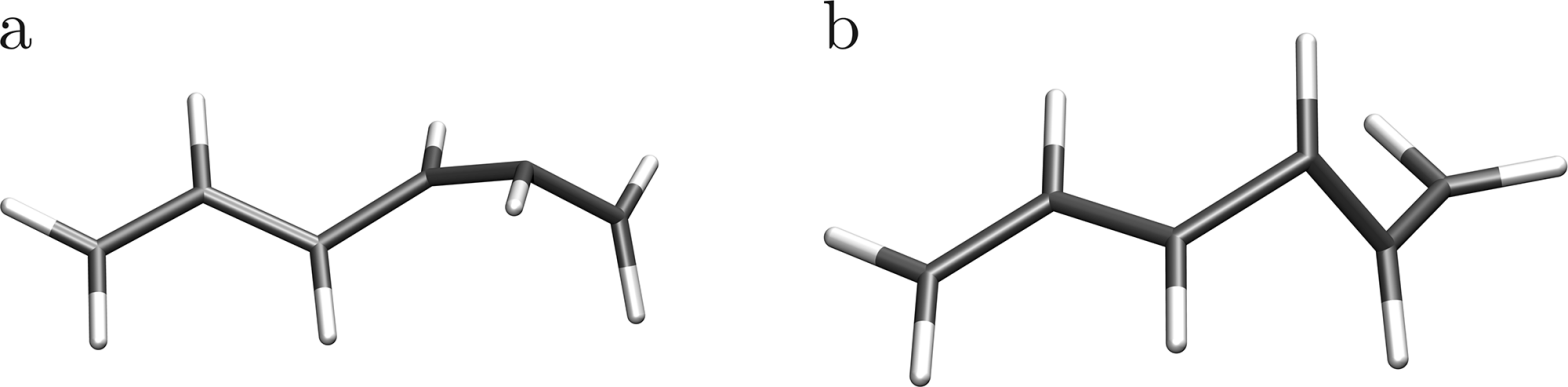
(a) MXS_1_ and (b) MXS_2_ structures. Geometries were optimized at the MR-CISD/SA2-CAS­(6,6)/cc-pVDZ level.

It should be emphasized that the seam of intersection in hexatriene spans a 34-dimensional subspace, and other structures along the seam are expected to contribute to the overall relaxation pathway.^69,70^ However, a full investigation of the different MXS rotamers at the MR-CISD level would be too time-consuming and is beyond the scope of the present investigation.

Efforts were also made to locate the MXS between S_2_ and S_1_ at the MR-CISD level. However, the energy separation between S_2_ and S_1_ was found to be systematically overestimated in the absence of size-extensivity corrections, hindering the convergence of a well-defined MXS optimization at this level.

The MXS_1_ interpolation path along the structures listed in Figure 3 is shown in Figure 5. It displays a smooth energetic evolution from the Franck–Condon region to the S_1_ minimum. The torsional motion toward MXS_1_ involves a small barrier, for which sufficient energy should be available for its crossing. A similar picture has been presented in ref. 15 based on CASSCF calculations. Figure S6 shows the analogous interpolation path to MXS_2_. Our MR-CISD+P calculations show that MXS_1_ (the lowest crossing point) lies approximately 0.14 eV below the S_1_ minimum (Figure 3, Table S5). Without size-extensivity corrections, it has nearly the same energy as the S_1_ minimum (Table S5). Its structure deviates strongly from planarity (Figure 6a) and exhibits appreciable radical character, mostly concentrated in the π frontier orbitals (Figure S5). Crucially, the distortion of the molecular backbone occurs in an asymmetric fashion: one terminal remains nearly coplanar, whereas the other exhibits a pronounced out-of-plane displacement.

**Figure 5:**
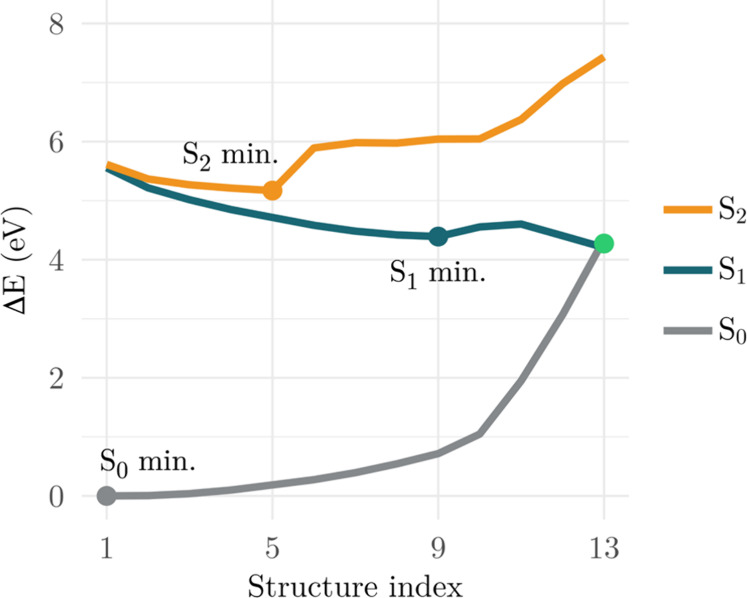
Potential energy profiles of the S_0_, S_1_, and S_2_ electronic states along a composite photochemical pathway constructed using geodesic interpolation. Single-point energies were calculated at the MR-CISD+P/SA6-CAS­(6,6)/cc-pVDZ level. The path consists of concatenated segments connecting the ground-state minimum to the S_2_ minimum, the S_2_ minimum to the S_1_ minimum, and finally the S_1_ minimum to the S_1_/S_0_ MXS_1_. Energies are reported relative to the ground-state minimum.

**Figure 6:**
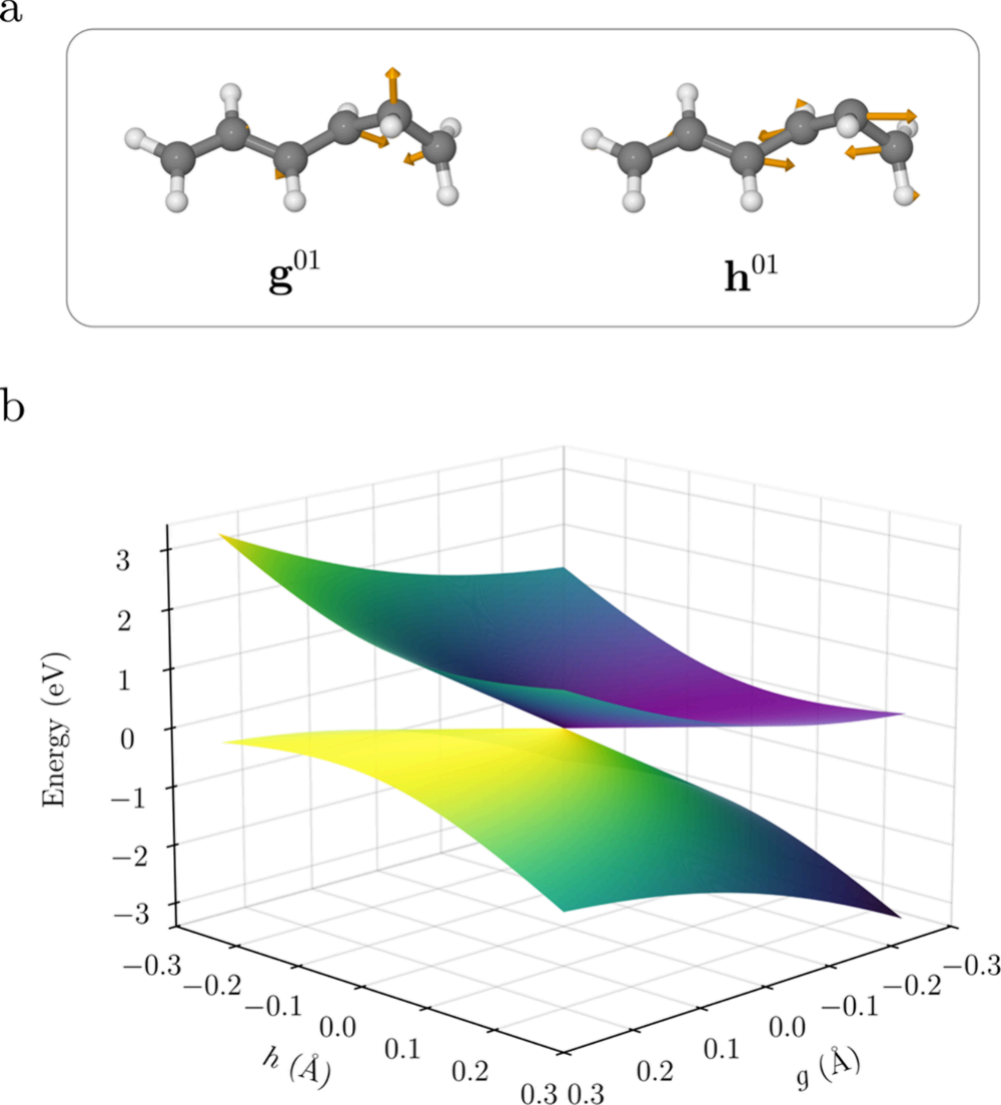
(a) Difference gradient vector **g** ^01^ and nonadiabatic coupling vector **h** ^01^ at the minimum of the crossing seam (MXS_1_) optimized at the MR-CISD/SA2-CAS­(6,6)/cc-pVDZ level. (b) Linearized adiabatic energies of the MXS in the g-h space.

The topography of the potential energy surfaces in the vicinity of the MXS plays an important role in determining its efficiency in facilitating nonadiabatic transitions.^71^ This region is conveniently characterized in terms of a set of scaled, orthogonal intersection-adapted coordinates that define the branching plane (or *g*–*h* plane), where the degeneracy between the intersecting electronic states is lifted linearly along the nuclear displacement coordinates *g* and *h*.^72,73^ The branching plane was defined by the Schmidt-orthogonalized energy difference gradient (**g** ^01^) and interstate coupling (**h** ^01^) vectors between the crossing states S_0_ and S_1_.^74–76^ The **g** ^01^ vector primarily corresponds to stretching and bending backbone motion consistent with bond-length alternation along the chain, whereas the **h** ^01^ vector is dominated by substantial out-of-plane components, characterizing a local pyramidalization (Figure 6a).

Here, we focus on analyzing the lowest crossing (MXS_1_). The local topographical features of the potential energy surfaces near the MXS can be described in terms of four conical parameters (eq S1a-S 1d). These parameters (*d*_*gh*_ = 6.71 eV · Å^–1^, Δ*_gh_* = −0.24, σ_*x*_ = 3.54, and σ_*y*_ = −2.23), as well as the linear approximation for the adiabatic energies of the crossing states, were calculated based on Equations S1–S4.^76^ The linearized adiabatic energies of the MXS in the *g*-*h* space are shown in Figure 6b. The four conical parameters indicate a steep and moderately asymmetric tilted conical intersection, suggesting that internal conversion through this and possibly other nearby intersections is extremely efficient. The large pitch (*d*_*gh*_ = 6.71 eV · Å^–1^) corresponds to steep potential energy surfaces in the branching plane, indicative of efficient population transfer between the intersecting electronic states. The small asymmetry parameter (Δ*_gh_* = −0.24) only slightly distorts the circular symmetry of the intersection. These two parameters govern the singularity in the derivative coupling that, in turn, drives nonadiabatic transitions.^77^ On the other hand, the substantial tilt parameters (σ_*x*_ = 3.54, and σ_*y*_ = −2.23) indicate a pronounced inclination of the cone axis, which has been shown to have no direct influence on the transition probability.^78^ A similar analysis applies to the higher energy MXS_2_ (Table S7).

The close proximity to the minimum of the S_1_ state, together with the topology of the potential energy surfaces near the crossing points, shows that the MXS structures act as efficient funnels for internal conversion to the ground state. This result is consistent with the ultrafast relaxation dynamics inferred from the absence of detectable fluorescence yields^40^ and the diffuse absorption spectra observed experimentally,^79^ as well as with the short lifetime (∼270 fs) of this state.^42^

Finally, for documentation purposes, the norms of the nonadiabatic coupling (NAC) vectors evaluated at the 1^1^A_g_^–^ (S_0_) equilibrium geometry at both the CASSCF and MR-CISD levels of theory are collected in Table S8. The analysis shows that dynamic correlation can significantly affect the magnitude of the NAC vectors in certain cases.

## 4 Conclusions

In this work, an accurate and balanced description of the ground state and the two lowest-lying excited states (2^1^A_g_^–^ and 1^1^B_u_^+^) in hexatriene, octatetraene, and decapentaene was achieved at the MR-CISD level, providing interesting insights into the spectroscopic behavior of polyenes and bridging theoretical predictions with experimental spectroscopy. Adiabatic excitation energies for the 2^1^A_g_^–^ states were calculated with excellent accuracy (within 0.1 eV) relative to the experimental absorption origin. Slightly larger, though still reasonable deviations (0.2–0.4 eV) were observed for the ionic 1^1^B_u_^+^ states, reflecting the challenges of describing this state at the multireference level due to the interplay of σ-π electron correlation and high-order excitations. Nonetheless, the high accuracy achieved for adiabatic excitation energies underscores the robustness of the MR-CISD wave function in providing reliable analytic energy gradients and nonadiabatic coupling vectors.

Our findings indicate that vertical excitation energies from the ground state to the 1^1^B_u_^+^ state are blue-shifted by 0.2–0.3 eV relative to the absorption band maximum. Vertical emission energies corresponding to radiative decay to the ground state were also computed. These results show that vertical emission from the 2^1^A_g_^–^ state is red-shifted by approximately 0.2 eV relative to the experimental emission maximum. Our results also indicate that, in emission from the 1^1^B_u_^+^ state, a small difference of approximately 0.3 eV between vertical excitation and vertical emission energies is observed, which is consistent with the small Stokes shift observed experimentally in octatetraene.

Upon geometry relaxation from the Franck–Condon structure, the 2^1^A_g_^–^ state is stabilized by about 1 eV, whereas the bright 1^1^B_u_^+^ state is stabilized by only 0.2–0.3 eV. This difference in relaxation energy is closely related to the distinct nuclear equilibrium geometries of the states involved. While the 2^1^A_g_^–^ state exhibits an alternating single–double bond pattern opposite to that of the ground state, the 1^1^B_u_^+^ state features a more equalized bond-length distribution along the polyene chain.

Finally, the S_1_/S_0_ MXS in hexatriene was characterized at the MR-CISD level. Two MXS structures are reported. The results show that the lowest conical intersection is located about 0.14 eV below the S_1_ C_2h_ minimum and involves strong nontotally symmetric deformations of the molecular backbone. The topology of the potential energy surfaces in the vicinity of the crossing point indicates that both MXS stru provides an efficient decay channel of decay to the ground state, which is consistent with the experimental observations of ultrafast relaxation dynamics, and the absence of detectable fluorescence yields−an aspect in which hexatriene differs from the larger polyenes studied. Taken together, these results demonstrate the reliability and computational efficiency of the MR-CISD and MR-CISD+P methods for elucidating the electronic structure and photophysics of conjugated polyenes.

## Supplementary Material



## Notes

Numerical data for adiabatic and vertical excitation energies, and relaxation energies; electronic configuration analysis; definitions of topographic parameters; gradient and nonadiabatic coupling vectors; equilibrium geometries (PDF)
