# Supplementary material for: Low-Lying Excited States of Linear All-Trans Polyenes: Insights from Analytic Gradient and Nonadiabatic Coupling Calculations Based on Multireference Configuration Interaction
Source: J Chem Theory Comput. 2026 May 4;22(10):4995–5004. doi: 10.1021/acs.jctc.6c00075 (PMC13217566; doi:10.1021/acs.jctc.6c00075)
Supplement: Supplementary file 1 [file ct6c00075_si_001.pdf]

Supplementary Information

Low-Lying Excited States of Linear All-*Trans* polyenes: Insights from Analytic Gradient and Nonadiabatic Coupling Calculations Based on Multireference Configuration Interaction

Julio C. V. Chagas,[1,2,3] Luan G. F. dos Santos,[4] Silmar A. do Monte,[5]
Adélia J. A. Aquino,[6] Felix Plasser,[7] Ron Shepard,[8] Péter G. Szalay,[9]
Hans Lischka,[4,*] and Franciso B. C. Machado[1,2,*]

*E-mail: hans.lischka@ttu.edu
*E-mail: fmachado@ita.br

[1] Department of Chemistry, Aeronautics Institute of Technology, São José dos Campos, SP 12228-900, Brazil
[2] Advanced Scientific Computing and Modeling Laboratory, Aeronautics Institute of Technology, São José dos Campos, SP 12228-900, Brazil
[3] Department of Chemistry, Northwestern University, Evanston, IL 60208, United States
[4] Department of Chemistry and Biochemistry, Texas Tech University, Lubbock, TX 79409, United States
[5] Department of Chemistry, Federal University of Paraíba, João Pessoa, PB 58059-900, Brazil
[6] Department of Mechanical Engineering, Texas Tech University, Lubbock, TX 79409, United States
[7] Department of Chemistry, Loughborough University, Loughborough, LE11 3TU, United Kingdom
[8] Chemical Sciences and Engineering Division, Argonne National Laboratory, Lemont, IL 60439, United States
[9] Institute of Chemistry, ELTE Eötvös Loránd University, Budapest, H-1117, Hungary

Contents

1 Adiabatic Excitation Energies **S2**

2 Vertical Excitation Energies **S2**

3 Relaxation Energies **S3**

4 Equilibrium Geometries **S5**
4.1 CC bond lengths S5

5 The Minimum of the Crossing Seam **S7**

6 Cartesian Coordinates **S13**
6.1 Hexatriene S13
6.2 Octatetraene S14
6.3 Decapentaene S15

References **S16**

1 Adiabatic Excitation Energies

Based on the optimized geometry of each state obtained via analytical energy gradients based on the MR-CISD wavefunction using the cc-pVDZ basis set, MR-CISD+P single point calculations were performed to analyze the effect of the basis set on adiabatic excitation energies (Table S1).

Table S1. Adiabatic excitation energies (eV) of polyenes with N π electrons computed at the MR-CISD+P level. Values computed using the cc-pVDZ basis set (ΔE_D), cc-pVTZ basis set (ΔE_T), as well as values extrapolated from double-zeta to triple-zeta (ΔE^{∞}_{DT}), are presented. All values are based on the minimum of each state obtained via analytical energy gradients based on the MR-CISD wavefunction using the cc-pVDZ basis set.

N	State	ΔE_D	ΔE_T	ΔE^{∞}_{DT}
6	$2^1A_g^-$	4.365	4.439	4.471
	$1^1B_u^+$	5.335	5.239	5.199
	$2^1B_u^-$	5.747	5.827	5.861
8	$2^1A_g^-$	3.645	3.718	3.748
	$1^1B_u^+$	4.698	4.761	4.787
	$2^1B_u^-$	5.038	5.129	5.168
10	$2^1A_g^-$	3.122	3.193	3.222
	$1^1B_u^+$	4.236	4.205	4.192
	$2^1B_u^-$	4.389	4.483	4.522

2 Vertical Excitation Energies

Based on the optimized geometry of the ground state obtained via analytical energy gradients based on the MR-CISD wavefunction using the cc-pVDZ basis set, MR-CISD+P single point calculations were performed to analyze the effect of the basis set on vertical excitation energies (Table S2).

Table S2. Vertical excitation energies (eV) of singlet states in polyenes with N π-electrons computed at the MR-CISD+P level. The ground-state geometry was optimized via analytic energy gradients based on the MR-CISD wavefunction using the cc-pVDZ basis set. Reference theoretical values and experimental data are provided for comparison.

N	State	MR-CISD+P			CASPT2	NEVPT2	Exp.
		ΔE_D	ΔE_T	ΔE^{∞}_{DT}			
6	$2^1A_g^-$	5.422	5.382	5.365	5.420[a], 5.09[b]	5.552, 5.517[d]	-
	$1^1B_u^+$	5.635	5.499	5.442	5.363[a], 5.10[b]	5.615, 5.478[d]	4.93 [f]
	$2^1B_u^-$	6.596	6.535	6.510	6.478[a]	6.518, 6.520[d]	–
8	$2^1A_g^-$	4.648	4.623	4.613	4.46[c], 4.47[b]	4.85, 4.81[e]	4.1 [g]
	$1^1B_u^+$	4.968	4.867	4.825	4.35[c], 4.66[b]	4.92, 4.76[e]	4.41 [h]
	$2^1B_u^-$	5.848	5.819	5.807	5.63[c]	5.98, 5.96[e]	–
10	$2^1A_g^-$	4.087	4.066	4.057	3.65[b]		3.5 [g]
	$1^1B_u^+$	4.479	4.398	4.364	4.05[b]		3.98 [i]
	$2^1B_u^-$	5.168	5.153	5.147	–		–

[a] Ground-state geometry optimized at the CAS(6,6)/NEVPT2 level; single-point energy at the CAS(6,14)/CASPT2 level.[1]
[b] Ground-state geometry optimized at the CAS(N,N)/CASSCF level; single-point energy at the CAS(N,N)/CASPT2 level, where N is the number of carbon atoms.[2]
[c] Ground-state geometry optimized at the CAS(8,8)/CASSCF level; single-point energy at the CAS(8,16)/CASPT2 level.[3]
[d] Ground-state geometry optimized at the CAS(6,6)/NEVPT2 level; single-point energy at the SC and PC CAS(6,14)/NEVPT2 levels, respectively.[1]
[e] Ground-state geometry optimized at the CAS(8,8)/NEVPT2 level; single-point energy at the SC and PC CAS(8,16)/NEVPT2 levels, respectively.[3]
[f] Absorption band maximum of the spectrum.[4,5]
[g] Estimated assuming mirror symmetry between absorption and emission. 0-0 excitation energy from Table 2 and emission band maximum from Table 3.
[h] Absorption band maximum of the spectrum.[6,7,8,9]
[i] Absorption band maximum in condensed phase, corrected to gas phase using solvent shift theory.[10]

The accuracy of experimental data for each state differs significantly due to their accessibility in one-photon processes. Experimental estimates for the vertical excitation energies of the $2^1A_g^-$ state rely on the mirror-image symmetry assumption, which posits that the energy difference between the (0-0) and vertical excitation energies is equal to the difference between the (0-0) and fluorescence intensity maximum—i.e., vertical excitation energies are estimated as $(0-0)+[(0-0)-\lambda_{emi}^{max}]$. On the other hand, for the $1^1B_u^+$ state, which is dipole-allowed and easily accessible in one-photon processes, the 0-0 excitation band also corresponds to the most intense absorption band.[11] We can also estimate the vertical excitation energy based on a mirror-image approximation using the MR-CISD+P values for adiabatic excitation (Table 2) and vertical emission energy (Table 3). This yields values of 5.10 eV for hexatriene, 4.33 eV for octatetraene, and 3.74 eV for decapentaene. For the two largest members, this represents an overestimation of approximately 0.2 eV with respect to the experimental estimate obtained via the mirror-image approximation.

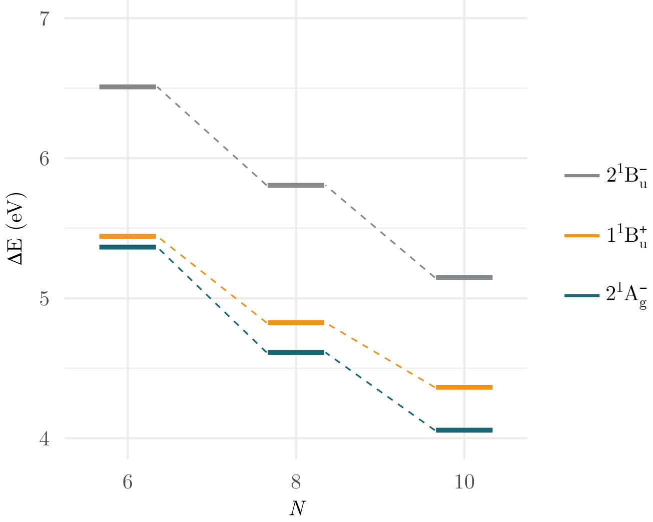

Figure S1. Vertical excitation energies (eV) of singlet states in polyenes with N π-electrons computed at the MR-CISD+P level extrapolated to complete basis set limit (ΔE_{DT}^{∞}). The ground-state geometry was optimized via analytic energy gradients based on the MR-CISD wavefunction using the cc-pVDZ basis set. Numerical data from Table S2.

3 Relaxation Energies

The relaxation energy is defined as the energy difference between vertical excitation energies (numerical data from Table S2) and adiabatic excitation energies (numerical data from Table S1).

Table S3. Relaxation energies (eV) of polyenes with N π electrons computed at the MR-CISD+P level. Values computed using the cc-pVDZ basis set (ΔE_D), cc-pVTZ basis set (ΔE_T), as well as values extrapolated from double-zeta to triple-zeta (ΔE_{DT}^{∞}), are presented. All values are based on the minimum of each state obtained via analytical energy gradients based on the MR-CISD wavefunction using the cc-pVDZ basis set.

N	State	ΔE_D	ΔE_T	ΔE_{DT}^{∞}
6	$2^1A_g^-$	1.057	0.942	0.894
	$1^1B_u^+$	0.300	0.260	0.243
	$2^1B_u^-$	0.849	0.708	0.649
8	$2^1A_g^-$	1.004	0.905	0.864
	$1^1B_u^+$	0.270	0.107	0.038
	$2^1B_u^-$	0.811	0.690	0.639
10	$2^1A_g^-$	0.965	0.873	0.835
	$1^1B_u^+$	0.243	0.193	0.172
	$2^1B_u^-$	0.779	0.671	0.626

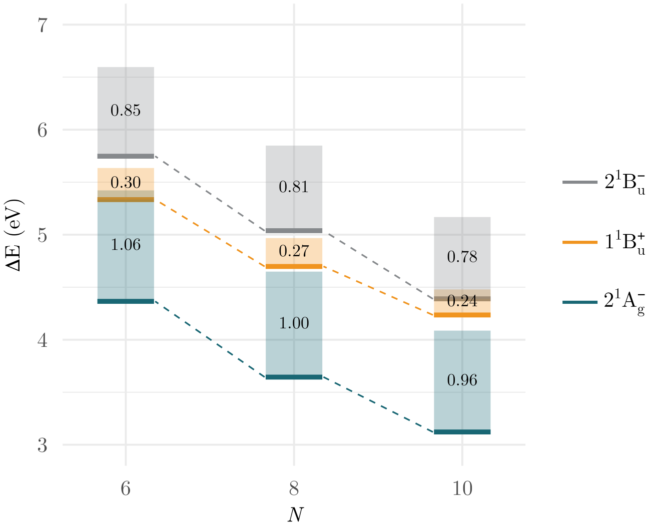

Figure S2. Relaxation energy (eV) in polyenes with N π-electrons computed at the MR-CISD+P level using the cc-pVDZ basis set. Relaxation energies-represented by the bar size and numeric values—is defined as the energy difference between vertical excitation energies (top of the bar) and adiabatic excitation energies (bottom of the bar). Numerical data from Table S3.

4 Equilibrium Geometries

4.1 CC bond lengths

The carbon numbering system is shown in Figure S5. The CC bond lengths of the studied systems in the ground and excited states, at their relaxed geometries obtained via analytical energy gradients based on the MR-CISD wavefunction, are presented in Table S8.

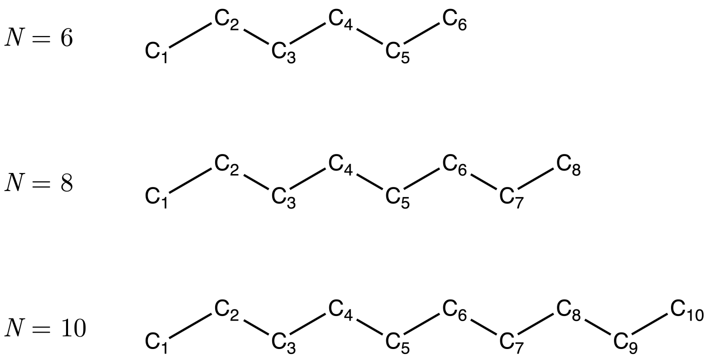

Figure S3. Atom numbering system used to label bonds in the studied polyenes with N π-electrons.

Table S4. Calculated CC bond lengths (Å) in polyenes with N π-electrons in the ground and excited states at their relaxed geometries, obtained via analytical energy gradients based on the MR-CISD wavefunction under the C_{2h} symmetry constraint. Atom numbering follows Figure S5. Values within parenthesis obtained at the CASSCF level.[2]

State	N	Bond				
		C_1-C_2	C_2-C_3	C_3-C_4	C_4-C_5	C_5-C_6
$1^1A_g^-$	6	1.353 (1.338)	1.458 (1.469)	1.360 (1.345)	–	–
	8	1.351 (1.345)	1.454 (1.457)	1.361 (1.351)	1.450 (1.451)	–
	10	1.350 (1.346)	1.450 (1.454)	1.359 (1.351)	1.445 (1.450)	1.362 (1.352)
$2^1A_g^-$	6	1.468 (1.445)	1.390 (1.383)	1.447 (1.427)	–	–
	8	1.439 (1.432)	1.380 (1.369)	1.441 (1.432)	1.396 (1.390)	–
	10	1.413 (1.407)	1.381 (1.375)	1.439 (1.434)	1.389 (1.385)	1.426 (1.415)
$1^1B_u^+$	6	1.392 (1.395)	1.410 (1.397)	1.423 (1.423)	–	–
	8	1.377 (1.379)	1.417 (1.413)	1.407 (1.401)	1.400 (1.395)	–
	10	1.370 (1.369)	1.422 (1.430)	1.394 (1.406)	1.400 (1.406)	1.403 (1.425)
$2^1B_u^-$	6	1.428	1.428	1.519	–	–
	8	1.429	1.428	1.452	1.362	–
	10	1.421	1.412	1.408	1.383	1.483

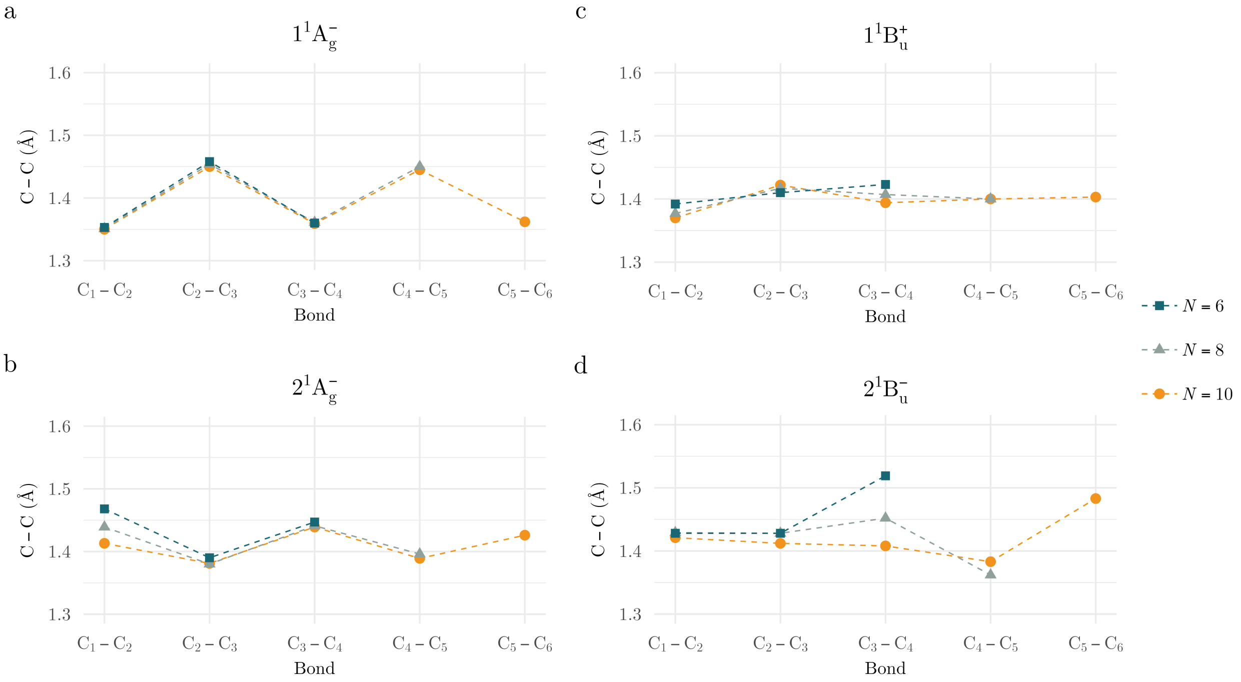

Figure S4. C-C bond lengths of the optimized geometries obtained via analytic energy gradients based on the MR-CISD wavefunction for the states: (a) $1^1A_g^-$, (b) $2^1A_g^-$, (c) $1^1B_u^+$, and (d) $2^1B_u^-$ of polyenes with N π-electrons. Numerical data from Table S8.

5 The Minimum of the Crossing Seam

Table S5. Total energies (a.u.) of the $1^1A_g^-$ (S_0), $2^1A_g^-$ (S_1), and $1^1B_u^+$ (S_2) states of hexatriene, computed at the CASSCF, MR-CISD, and MR-CISD+P levels. The energy difference (eV) with respect to the minimum of the ground state is also given. The minimum of the S_0/S_1 crossing seam (MXS) is also indicated. All values are obtained based on MR-CISD+P/SA6-CAS(6,6)/cc-pVDZ single point calculations based on optimized geometries obtained via analytic energy gradients and nonadiabatic coupling vectors based on the MR-CISD wavefunction, as indicated in the main text.

		Total Energy (a.u.)			**ΔE to S_0 min. (eV)**		
**Geometry**	**State**	CASSCF	MR-CISD	MR-CISD+P	CASSCF	MR-CISD	MR-CISD+P
S_0 min.	S_0	-231.90529	-232.55813	-232.70795	0.000	0.000	0.000
	S_1	-231.70571	-232.35727	-232.50871	5.431	5.466	5.422
	S_2	-231.63059	-232.32412	-232.50086	7.475	6.368	5.635
S_1 min.	S_0	-231.87434	-232.52786	-232.68092	0.842	0.824	0.735
	S_1	-231.74613	-232.39601	-232.54754	4.331	4.412	4.365
	S_2	-231.62734	-232.32371	-232.50521	7.563	6.379	5.517
S_2 min.	S_0	-231.89657	-232.54983	-232.70078	0.237	0.226	0.195
	S_1	-231.73488	-232.38525	-232.53585	4.637	4.704	4.683
	S_2	-231.63951	-232.33408	-232.51190	7.232	6.097	5.335
MXS_1	S_0/S_1	-231.74076	-232.39611	-232.55265	4.477	4.409	4.226
	S_0/S_1	-231.73316	-232.39489	-232.55733	4.684	4.442	4.098
MXS_2	S_0/S_1	-231.73862	-232.39394	-232.55003	4.535	4.468	4.297
	S_0/S_1	-231.72955	-232.39169	-232.55483	4.782	4.529	4.167

Table S6. Dominant electronic configurations and orbital excitations of the $1^1A_g^-$ (S0), 2^1Ag^- (S1), and 1^1Bu^+ (S_2) states of hexatriene, computed at the MR-CISD level at the minimum of each electronic state. The electronic configuration at the minimum of the S_0/S_1 crossing seam (MXS) is also indicated.

Geometry	State	f	Electron Configuration	Excitation	Contribution (c^2)
S_0 min.	S_0	–	$(1a_u)^2\ (2a_u)^2\ (3a_u)^0\ (1b_g)^2\ (2b_g)^0\ (3b_g)^0$	Reference	0.729
	S_1	0.000	$(1a_u)^2\ (2a_u)^0\ (3a_u)^0\ (1b_g)^2\ (2b_g)^2\ (3b_g)^0$	$(2a_u)^2 \rightarrow (2b_g)^2$	0.273
			$(1a_u)^2\ (2a_u)^2\ (3a_u)^0\ (1b_g)^1\ (2b_g)^1\ (3b_g)^0$	$(1b_g)^1 \rightarrow (2b_g)^1$	0.211
			$(1a_u)^2\ (2a_u)^1\ (3a_u)^1\ (1b_g)^2\ (2b_g)^0\ (3b_g)^0$	$(2a_u)^1 \rightarrow (3a_u)^1$	0.155
	S_2	1.262	$(1a_u)^2\ (2a_u)^1\ (3a_u)^0\ (1b_g)^2\ (2b_g)^1\ (3b_g)^0$	$(2a_u)^1 \rightarrow (2b_g)^1$	0.736
S_1 min.	S_0		$(1a_u)^2\ (2a_u)^2\ (3a_u)^0\ (1b_g)^2\ (2b_g)^0\ (3b_g)^0$	Reference	0.675
	S_1		$(1a_u)^2\ (2a_u)^0\ (3a_u)^0\ (1b_g)^2\ (2b_g)^2\ (3b_g)^0$	$(2a_u)^2 \rightarrow (2b_g)^2$	0.275
			$(1a_u)^2\ (2a_u)^2\ (3a_u)^0\ (1b_g)^1\ (2b_g)^1\ (3b_g)^0$	$(1b_g)^1 \rightarrow (2b_g)^1$	0.197
			$(1a_u)^2\ (2a_u)^1\ (3a_u)^1\ (1b_g)^2\ (2b_g)^0\ (3b_g)^0$	$(2a_u)^1 \rightarrow (3a_u)^1$	0.151
	S_2		$(1a_u)^2\ (2a_u)^1\ (3a_u)^0\ (1b_g)^2\ (2b_g)^1\ (3b_g)^0$	$(2a_u)^1 \rightarrow (2b_g)^1$	0.701
S_2 min.	S_0		$(1a_u)^2\ (2a_u)^2\ (3a_u)^0\ (1b_g)^2\ (2b_g)^0\ (3b_g)^0$	Reference	0.709
	S_1		$(1a_u)^2\ (2a_u)^0\ (3a_u)^0\ (1b_g)^2\ (2b_g)^2\ (3b_g)^0$	$(2a_u)^2 \rightarrow (2b_g)^2$	0.285
			$(1a_u)^2\ (2a_u)^2\ (3a_u)^0\ (1b_g)^1\ (2b_g)^1\ (3b_g)^0$	$(1b_g)^1 \rightarrow (2b_g)^1$	0.202
			$(1a_u)^2\ (2a_u)^1\ (3a_u)^1\ (1b_g)^2\ (2b_g)^0\ (3b_g)^0$	$(2a_u)^1 \rightarrow (3a_u)^1$	0.152
	S_2		$(1a_u)^2\ (2a_u)^1\ (3a_u)^0\ (1b_g)^2\ (2b_g)^1\ (3b_g)^0$	$(2a_u)^1 \rightarrow (2b_g)^1$	0.716
MXS_1	S_0/S_1		$(20a)^2\ (21a)^2\ (22a)^2\ (23a)^0\ (24a)^0\ (25a)^0$	$(22a)^1 \rightarrow (23a)^1$	0.445
			$(20a)^2\ (21a)^2\ (22a)^1\ (23a)^1\ (24a)^0\ (25a)^0$		0.118
	S_0/S_1		$(20a)^2\ (21a)^2\ (22a)^1\ (23a)^1\ (24a)^0\ (25a)^0$	$(22a)^1 \rightarrow (23a)^1$	0.470
			$(20a)^2\ (21a)^2\ (22a)^2\ (23a)^0\ (24a)^0\ (25a)^0$		0.116
MXS_2	S_0/S_1		$(20a)^2\ (21a)^2\ (22a)^2\ (23a)^0\ (24a)^0\ (25a)^0$	$(22a)^1 \rightarrow (23a)^1$	0.357
			$(20a)^2\ (21a)^2\ (22a)^1\ (23a)^1\ (24a)^0\ (25a)^0$		0.234
	S_0/S_1		$(20a)^2\ (21a)^2\ (22a)^1\ (23a)^1\ (24a)^0\ (25a)^0$	$(22a)^1 \rightarrow (23a)^1$	0.368
			$(20a)^2\ (21a)^2\ (22a)^2\ (23a)^0\ (24a)^0\ (25a)^0$		0.226

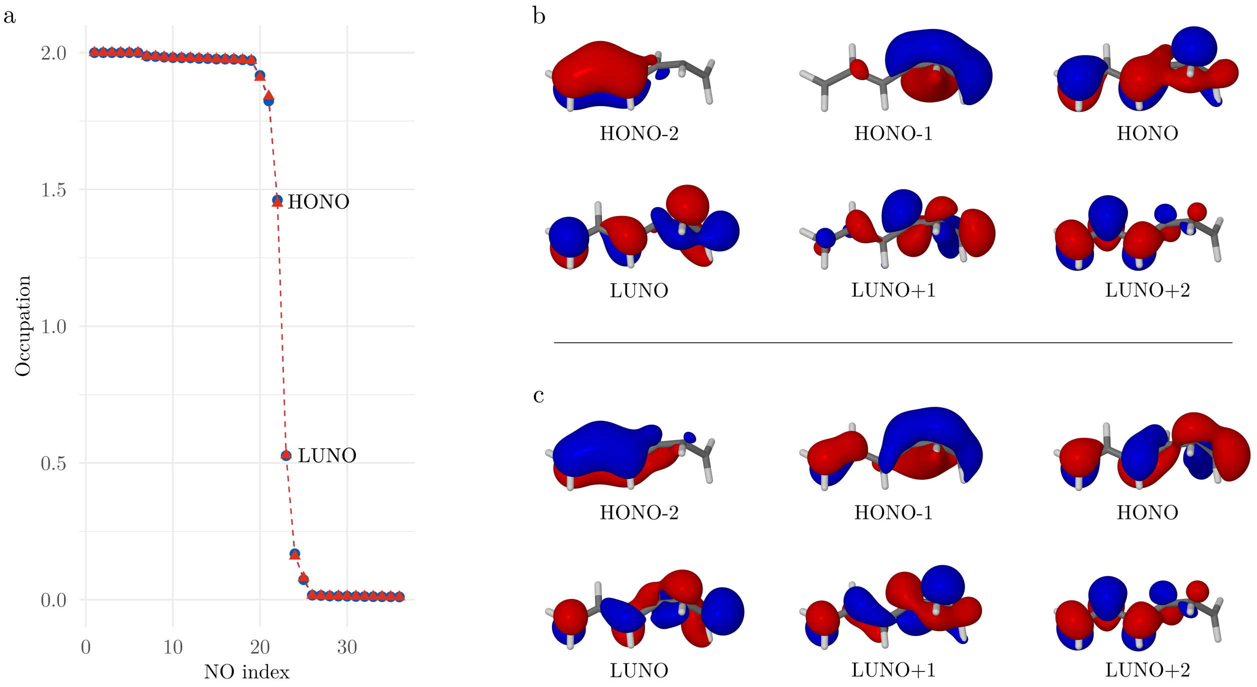

Figure S5. (a) Natural orbital occupation numbers for the two crossing states at the minimum of the lowest S_0/S_1 crossing seam (MXS_1). (b) and (c) show the open-shell natural orbitals of the two crossing states.

The topographic features of the minimum of the crossing seam (MXS) can be described in terms of four parameters:[12]

$$\sigma_x = \frac{\mathbf{s}^{01} \cdot \hat{\mathbf{x}}}{d_{gh}} \tag{1a}$$

$$\sigma_y = \frac{\mathbf{s}^{01} \cdot \hat{\mathbf{y}}}{d_{gh}} \tag{1b}$$

$$\Delta_{gh} = \frac{g^2 - h^2}{d_{gh}^2} \tag{1c}$$

$$d_{gh} = (g^2 + h^2)^{1/2} \tag{1d}$$

where $\mathbf{s}^{01}$ is the gradient sum vector, and $\hat{\mathbf{x}}$ and $\hat{\mathbf{y}}$ are unit vectors based on the Schmidt-orthogonalized difference gradient vector $\mathbf{g}$ and nonadiabatic coupling vector $\mathbf{h}$:

$$\hat{\mathbf{x}} = \mathbf{g}^{01}/g, \quad g = \|\mathbf{g}^{01}\| \tag{2}$$

$$\hat{\mathbf{y}} = \mathbf{h}^{01}/h, \quad h = \|\mathbf{h}^{01}\| \tag{3}$$

The linear approximation for the adiabatic energies of the crossing states is given by

$$E = d_{gh}\left[\sigma_x x + \sigma_y y \pm \left(\frac{1}{2}(x^2 + y^2) + \frac{\Delta_{gh}}{2}(x^2 - y^2)\right)^{1/2}\right]. \tag{4}$$

Table S7. Conical parameters d_{gh}, Δ_{gh}, σ_x, and σ_y that describe local topographical features of the potential energy surfaces near MXS_1 and MXS_2.

MXS	d_{gh}	Δ_{gh}	σ_x	σ_y
MXS_1	6.71	-0.24	3.54	-2.23
MXS_2	6.11	-0.36	-3.27	1.32

Geometries in cartesian coordinates, as well as orthogonalized $\mathbf{g}^{01}$ and $\mathbf{h}^{01}$ vectors for MXS_1 and MXS_2, are presented below.

MXS_1 S_0/S_1			
C	1.229578	2.566100	-0.142225
C	-0.978303	-2.809238	0.038333
C	0.959857	1.534987	0.874868
C	-1.041392	-1.432047	0.202992
C	-0.073513	0.894522	0.161092
C	-0.022109	-0.540036	-0.163250
H	1.959413	2.382189	-0.931363
H	-1.822294	-3.445129	0.288493
H	0.506475	3.360354	-0.321185
H	-0.086988	-3.284627	-0.365706
H	1.773689	0.942196	1.289274
H	-1.953763	-1.008124	0.625851
H	-0.941649	1.479858	-0.142515
H	0.909718	-0.935633	-0.564835

MXS_1 Othogonalized $\mathbf{g}^{01}$ vector		
-0.146743E-01	-0.262687E-01	-0.148763E-01
0.948623E-03	-0.537943E-02	0.371955E-03
-0.193511E-02	-0.307293E-02	0.491208E-01
0.854524E-02	0.106546E-01	0.963847E-02
0.114847E-01	0.373068E-01	-0.218400E-01
0.237912E-02	-0.155877E-01	-0.138865E-01
-0.534862E-02	-0.319647E-02	-0.627602E-02
-0.590881E-03	0.326170E-03	-0.173304E-02
0.233826E-02	0.348178E-02	0.232251E-02
-0.322327E-04	0.351231E-04	0.292869E-03
-0.754879E-03	0.653257E-03	0.177153E-03
-0.228209E-03	-0.159137E-04	-0.254885E-04
0.331275E-03	0.296460E-03	0.987385E-03
-0.246306E-02	0.766908E-03	-0.427379E-02

MXS_1 Othogonalized $\mathbf{h}^{01}$ vector		
-0.954892E-02	-0.454216E-01	0.437817E-02
-0.530641E-03	0.110676E-01	0.116880E-02
0.401554E-01	0.374483E-01	0.121224E-01
-0.119757E-01	-0.160716E-01	-0.436274E-02
-0.372177E-01	-0.225274E-01	-0.249725E-01
0.189440E-01	0.355292E-01	-0.704104E-02
0.929984E-02	0.848916E-02	0.381588E-02
0.808181E-04	0.140528E-03	0.796771E-03
-0.481452E-02	-0.391384E-02	-0.256065E-02
0.244975E-03	-0.697978E-04	0.183865E-03
0.105984E-02	0.982341E-04	-0.193370E-02
-0.304159E-03	-0.799043E-04	-0.183725E-03
-0.859625E-02	-0.291148E-02	0.122083E-01
0.320307E-02	-0.177754E-02	0.638024E-02

MXS$_2$ S$_0$/S$_1$			
C	-0.066939	0.080029	-0.069895
C	-0.697599	-1.130575	0.073162
C	-2.111665	-1.302128	0.024949
C	-2.798415	-2.585910	0.198717
C	-3.104731	-2.806804	-1.169169
C	-2.604201	-4.008350	-1.826259
H	1.008581	0.172809	0.051709
H	-0.629606	0.987151	-0.282787
H	-0.096310	-2.013076	0.295045
H	-2.717874	-0.442832	-0.268183
H	-2.330912	-3.384219	0.773989
H	-3.678010	-2.054396	-1.711036
H	-1.554850	-4.269354	-1.708814
H	-3.294422	-4.827066	-2.036786

MXS$_2$ Othogonalized $\mathbf{g}^{01}$ vector		
0.202664E-02	0.278151E-02	-0.951859E-03
-0.722558E-02	-0.115535E-02	0.116378E-01
0.281103E-02	0.903435E-02	0.226100E-01
0.289879E-01	0.226313E-01	-0.270009E-01
-0.152884E-01	-0.309345E-01	-0.348823E-03
-0.116135E-01	0.130045E-02	0.560835E-02
0.266235E-03	-0.309269E-03	-0.208151E-02
-0.507747E-05	-0.345241E-04	0.268610E-03
0.273403E-03	0.900229E-04	0.600666E-03
0.621916E-03	-0.117722E-02	-0.344687E-02
0.846262E-03	-0.169039E-03	-0.940186E-04
-0.246083E-02	-0.190123E-02	-0.557926E-03
-0.210166E-05	-0.273615E-02	0.653640E-02
0.762159E-03	0.257965E-02	-0.127798E-01

MXS$_2$ Othogonalized $\mathbf{h}^{01}$ vector		
0.747475E-02	0.112864E-01	-0.190965E-02
-0.244866E-01	-0.157004E-01	-0.631368E-02
0.173351E-01	0.403737E-01	0.124770E-01
-0.211734E-01	-0.284015E-01	-0.414513E-01
0.988841E-02	0.880754E-02	0.445758E-01
-0.697956E-02	-0.244528E-01	-0.982685E-02
-0.307261E-03	0.157625E-03	0.740664E-03
0.182733E-03	-0.141325E-03	0.213930E-03
0.717252E-03	-0.527910E-03	0.414033E-03
0.757543E-03	0.210499E-02	0.550633E-02
-0.766264E-03	-0.346256E-03	-0.312757E-04
0.152737E-01	0.675428E-02	-0.281301E-02
0.376254E-03	0.539402E-02	-0.105924E-01
0.170731E-02	-0.530832E-02	0.901038E-02

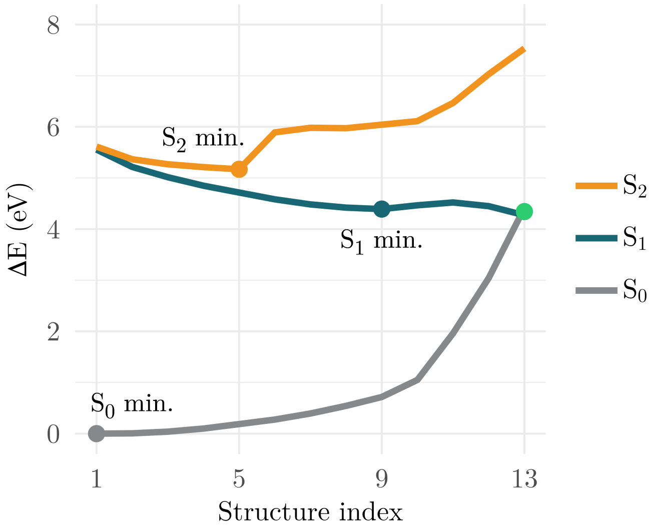

Figure S6. Potential energy profiles of the S_0, S_1, and S_2 electronic states along a composite photochemical pathway constructed using geodesic interpolation. The path consists of concatenated segments connecting the ground-state minimum to the S_2 minimum, the S_2 minimum to the S_1 minimum and finally the S_1 minimum to the S_1/S_0 MXS$_2$. Energies are reported relative to the ground-state minimum.

Table S8. Norms of the nonadiabatic coupling vectors $|\mathrm{h}|_{ij}$(a.u.) between selected pairs of electronic states i and j of all-trans-hexatriene, obtained at the CASSCF and MR-CISD levels of theory, evaluated at the S_0 equilibrium geometry.[a]

$\|\mathrm{h}_{ij}\|$	cc-pVDZ		aug-cc-pVDZ	
	CASSCF	MR-CISD	CASSCF	MR-CISD
$\|1^1A_g^-/2^1A_g^-\|$	0.751	0.733	0.657	0.668
$\|1^1A_g^-/1^1B_u^+\|$	0.283	0.273	0.327	0.297
$\|2^1A_g^-/1^1B_u^+\|$	0.193	0.441	0.547	2.875

[a] The ionic B_u state is consistently chosen; it corresponds to $2B_u$ at the CASSCF level and $1B_u$ at the MR-CI level.

6 Cartesian Coordinates

Equilibrium geometries (Å) of the states $1^1A_g^-$, $2^1A_g^-$, and $1^1B_u^+$ of hexatriene (Section 6.1), octatetraene (Section 6.2), and decapentaene (Section 6.3), obtained via analytical gradients based on the MR-CISD wavefunction, are presented below.

6.1 Hexatriene

$1^1A_g^-$			
C	1.212244	2.846199	0.000000
C	-1.212244	-2.846199	0.000000
C	1.209667	1.493113	0.000000
C	-1.209667	-1.493113	0.000000
C	-0.000222	0.679762	0.000000
C	0.000222	-0.679762	0.000000
H	2.141056	3.410545	0.000000
H	-2.141056	-3.410545	0.000000
H	0.282941	3.413640	0.000000
H	-0.282941	-3.413640	0.000000
H	2.160163	0.958195	0.000000
H	-2.160163	-0.958195	0.000000
H	-0.954033	1.210056	0.000000
H	0.954033	-1.210056	0.000000

$2^1A_g^-$			
C	1.233633	2.938021	0.000000
C	-1.233633	-2.938021	0.000000
C	1.193689	1.470122	0.000000
C	-1.193689	-1.470122	0.000000
C	0.021076	0.723259	0.000000
C	-0.021076	-0.723259	0.000000
H	2.180707	3.465976	0.000000
H	-2.180707	-3.465976	0.000000
H	0.313851	3.515505	0.000000
H	-0.313851	-3.515505	0.000000
H	2.146903	0.942481	0.000000
H	-2.146903	-0.942481	0.000000
H	-0.933263	1.251796	0.000000
H	0.933263	-1.251796	0.000000

$1^1B_u^+$			
C	1.218192	2.879558	0.000000
C	-1.218192	-2.879558	0.000000
C	1.194535	1.487753	0.000000
C	-1.194535	-1.487753	0.000000
C	0.017830	0.711052	0.000000
C	-0.017830	-0.711052	0.000000
H	2.158063	3.422557	0.000000
H	-2.158063	-3.422557	0.000000
H	0.296328	3.457034	0.000000
H	-0.296328	-3.457034	0.000000
H	2.146105	0.954783	0.000000
H	-2.146105	-0.954783	0.000000
H	-0.939243	1.235209	0.000000
H	0.939243	-1.235209	0.000000

$2^1B_u^-$			
C	1.259294	2.947951	0.000000
C	-1.259294	-2.947951	0.000000
C	1.199393	1.521220	0.000000
C	-1.199393	-1.521220	0.000000
C	-0.008801	0.759585	0.000000
C	0.008801	-0.759585	0.000000
H	2.214244	3.462655	0.000000
H	-2.214244	-3.462655	0.000000
H	0.351257	3.544759	0.000000
H	-0.351257	-3.544759	0.000000
H	2.140692	0.972923	0.000000
H	-2.140692	-0.972923	0.000000
H	-0.971166	1.265300	0.000000
H	0.971166	-1.265300	0.000000

6.2 Octatetraene

$1^1A_g^-$			
C	-0.000898	0.725026	0.000000
C	0.000898	-0.725026	0.000000
C	1.126034	1.487443	0.000000
C	-1.126034	-1.487443	0.000000
C	1.120340	2.941139	0.000000
C	-1.120340	-2.941139	0.000000
C	2.235944	3.702764	0.000000
C	-2.235944	-3.702764	0.000000
H	-0.972102	1.219853	0.000000
H	0.972102	-1.219853	0.000000
H	2.098279	0.994667	0.000000
H	-2.098279	-0.994667	0.000000
H	0.145078	3.426970	0.000000
H	-0.145078	-3.426970	0.000000
H	3.226755	3.254049	0.000000
H	-3.226755	-3.254049	0.000000
H	2.180137	4.786974	0.000000
H	-2.180137	-4.786974	0.000000

$2^1A_g^-$			
C	-0.018907	0.697631	0.000000
C	0.018907	-0.697631	0.000000
C	1.153315	1.535873	0.000000
C	-1.153315	-1.535873	0.000000
C	1.119347	2.914973	0.000000
C	-1.119347	-2.914973	0.000000
C	2.286381	3.757313	0.000000
C	-2.286381	-3.757313	0.000000
H	-0.990206	1.190646	0.000000
H	0.990206	-1.190646	0.000000
H	2.123120	1.038485	0.000000
H	-2.123120	-1.038485	0.000000
H	0.146787	3.405382	0.000000
H	-0.146787	-3.405382	0.000000
H	3.282008	3.325100	0.000000
H	-3.282008	-3.325100	0.000000
H	2.193969	4.836871	0.000000
H	-2.193969	-4.836871	0.000000

$1^1B_u^+$			
C	-0.008571	0.699762	0.000000
C	0.008571	-0.699762	0.000000
C	1.142383	1.509457	0.000000
C	-1.142383	-1.509457	0.000000
C	1.127138	2.926565	0.000000
C	-1.127138	-2.926565	0.000000
C	2.252001	3.720900	0.000000
C	-2.252001	-3.720900	0.000000
H	-0.977814	1.198178	0.000000
H	0.977814	-1.198178	0.000000
H	2.112351	1.012203	0.000000
H	-2.112351	-1.012203	0.000000
H	0.150922	3.410943	0.000000
H	-0.150922	-3.410943	0.000000
H	3.248495	3.286620	0.000000
H	-3.248495	-3.286620	0.000000
H	2.173945	4.802826	0.000000
H	-2.173945	-4.802826	0.000000

$2^1B_u^-$			
C	-0.004064	0.680881	0.000000
C	0.004064	-0.680881	0.000000
C	1.187185	1.511417	0.000000
C	-1.187185	-1.511417	0.000000
C	1.136581	2.938598	0.000000
C	-1.136581	-2.938598	0.000000
C	2.287104	3.786698	0.000000
C	-2.287104	-3.786698	0.000000
H	-0.964623	1.196509	0.000000
H	0.964623	-1.196509	0.000000
H	2.157957	1.020688	0.000000
H	-2.157957	-1.020688	0.000000
H	0.155686	3.409406	0.000000
H	-0.155686	-3.409406	0.000000
H	3.288435	3.368102	0.000000
H	-3.288435	-3.368102	0.000000
H	2.180526	4.864994	0.000000
H	-2.180526	-4.864994	0.000000

6.3 Decapentaene

$1^1A_g^-$			
C	2.416384	5.002357	0.000000
C	-2.416384	-5.002357	0.000000
C	2.407503	3.652414	0.000000
C	-2.407503	-3.652414	0.000000
C	1.201562	2.846971	0.000000
C	-1.201562	-2.846971	0.000000
C	1.198528	1.487893	0.000000
C	-1.198528	-1.487893	0.000000
C	0.000309	0.680776	0.000000
C	-0.000309	-0.680776	0.000000
H	3.346030	5.561345	0.000000
H	-3.346030	-5.561345	0.000000
H	1.491757	5.573495	0.000000
H	-1.491757	-5.573495	0.000000
H	3.354588	3.115529	0.000000
H	-3.354588	-3.115529	0.000000
H	0.250404	3.377439	0.000000
H	-0.250404	-3.377439	0.000000
H	2.151363	0.960277	0.000000
H	-2.151363	-0.960277	0.000000
H	-0.953089	1.207124	0.000000
H	0.953089	-1.207124	0.000000

$2^1A_g^-$			
C	2.435702	5.057419	0.000000
C	-2.435702	-5.057419	0.000000
C	2.386735	3.645427	0.000000
C	-2.386735	-3.645427	0.000000
C	1.224197	2.900480	0.000000
C	-1.224197	-2.900480	0.000000
C	1.189830	1.461896	0.000000
C	-1.189830	-1.461896	0.000000
C	0.019737	0.712862	0.000000
C	-0.019737	-0.712862	0.000000
H	3.382080	5.584119	0.000000
H	-3.382080	-5.584119	0.000000
H	1.524219	5.646151	0.000000
H	-1.524219	-5.646151	0.000000
H	3.333840	3.108202	0.000000
H	-3.333840	-3.108202	0.000000
H	0.269870	3.424637	0.000000
H	-0.269870	-3.424637	0.000000
H	2.142878	0.936197	0.000000
H	-2.142878	-0.936197	0.000000
H	-0.931644	1.242232	0.000000
H	0.931644	-1.242232	0.000000

$1^1B_u^+$			
C	2.422001	5.018231	0.000000
C	-2.422001	-5.018231	0.000000
C	2.393339	3.648412	0.000000
C	-2.393339	-3.648412	0.000000
C	1.206515	2.865054	0.000000
C	-1.206515	-2.865054	0.000000
C	1.184692	1.471007	0.000000
C	-1.184692	-1.471007	0.000000
C	0.015114	0.701291	0.000000
C	-0.015114	-0.701291	0.000000
H	3.361133	5.559740	0.000000
H	-3.361133	-5.559740	0.000000
H	1.505474	5.601302	0.000000
H	-1.505474	-5.601302	0.000000
H	3.339408	3.109365	0.000000
H	-3.339408	-3.109365	0.000000
H	0.253591	3.392427	0.000000
H	-0.253591	-3.392427	0.000000
H	2.138762	0.945738	0.000000
H	-2.138762	-0.945738	0.000000
H	-0.939000	1.226661	0.000000
H	0.939000	-1.226661	0.000000

$2^1B_u^-$			
C	2.461674	5.071922	0.000000
C	-2.461674	-5.071922	0.000000
C	2.400587	3.652588	0.000000
C	-2.400587	-3.652588	0.000000
C	1.202320	2.905977	0.000000
C	-1.202320	-2.905977	0.000000
C	1.158288	1.499098	0.000000
C	-1.158288	-1.499098	0.000000
C	0.001247	0.741543	0.000000
C	-0.001247	-0.741543	0.000000
H	3.413823	5.587519	0.000000
H	-3.413823	-5.587519	0.000000
H	1.555619	5.668147	0.000000
H	-1.555619	-5.668147	0.000000
H	3.338882	3.102431	0.000000
H	-3.338882	-3.102431	0.000000
H	0.259146	3.449319	0.000000
H	-0.259146	-3.449319	0.000000
H	2.111569	0.970966	0.000000
H	-2.111569	-0.970966	0.000000
H	-0.963342	1.243632	0.000000
H	0.963342	-1.243632	0.000000

References

[1] Riccardo Guareschi and Celestino Angeli. "The lowest singlet states of hexatriene revisited". In: *Theoretical Chemistry Accounts* 142 (12 Dec. 2023), p. 127. ISSN: 1432-881X. DOI: 10.1007/s00214-023-03064-y. URL: https://link.springer.com/10.1007/s00214-023-03064-y.

[2] Kenichi Nakayama, Haruyuki Nakano, and Kimihiko Hirao. "Theoretical study of the $\pi\pi^*$ excited states of linear polyenes: The energy gap between 11Bu+ and 21Ag states and their character". In: *International Journal of Quantum Chemistry* 66 (2 1998), pp. 157–175. ISSN: 0020-7608. DOI: 10.1002/(SICI)1097-461X(1998)66:2<157::AID-QUA7>3.0.CO;2-U. URL: https://onlinelibrary.wiley.com/doi/10.1002/(SICI)1097-461X(1998)66:2%3C157::AID-QUA7%3E3.0.CO;2-U.

[3] Celestino Angeli and Mariachiara Pastore. "The lowest singlet states of octatetraene revisited". In: *The Journal of Chemical Physics* 134 (18 May 2011). ISSN: 0021-9606. DOI: 10.1063/1.3585607. URL: https://pubs.aip.org/jcp/article/134/18/184302/189306/The-lowest-singlet-states-of-octatetraene.

[4] R. M. Gavin, Salomon Risemberg, and Stuart A. Rice. "Spectroscopic properties of polyenes. I. The lowest energy allowed singlet-singlet transition for cis - and trans - 1,3,5-hexatriene". In: *The Journal of Chemical Physics* 58 (8 Apr. 1973), pp. 3160–3165. ISSN: 0021-9606. DOI: 10.1063/1.1679637. URL: https://pubs.aip.org/jcp/article/58/8/3160/462854/Spectroscopic-properties-of-polyenes-I-The-lowest.

[5] D. G. Leopold et al. "Direct absorption spectroscopy of jet-cooled polyenes. II. The $1^1B_u^+ \leftarrow 1^1A_g^-$ transitions of butadienes and hexatrienes". In: *The Journal of Chemical Physics* 81.10 (Nov. 1984), pp. 4218–4229. ISSN: 0021-9606. DOI: 10.1063/1.447453. URL: https://pubs.aip.org/jcp/article/81/10/4218/90656/Direct-absorption-spectroscopy-of-jet-cooled.

[6] R. M. Gavin et al. "Spectroscopic properties of polyenes. III. 1,3,5,7-Octatetraene". In: *The Journal of Chemical Physics* 68 (2 Jan. 1978), pp. 522–529. ISSN: 0021-9606. DOI: 10.1063/1.435761. URL: https://pubs.aip.org/jcp/article/68/2/522/533096/Spectroscopic-properties-of-polyenes-III-1-3-5-7.

[7] Wim G. Bouwman et al. "Fluorescence of gaseous tetraenes and pentaenes". In: *The Journal of Physical Chemistry* 94.19 (Sept. 1990), pp. 7429–7434. ISSN: 0022-3654. DOI: 10.1021/j100382a022. URL: https://pubs.acs.org/doi/abs/10.1021/j100382a022.

[8] D. G. Leopold, V. Vaida, and Mark F. Granville. "Direct absorption spectroscopy of jet-cooled polyenes. I. The $1^1B_u^+ \leftarrow 1^1A_g^-$ transition of *trans*,trans-1,3,5,7-octatetraene". In: *The Journal of Chemical Physics* 81.10 (Nov. 1984), pp. 4210–4217. ISSN: 0021-9606. DOI: 10.1063/1.447452. URL: https://pubs.aip.org/jcp/article/81/10/4210/90893/Direct-absorption-spectroscopy-of-jet-cooled.

[9] Lou Ann Heimbrook, Bryan E. Kohler, and Irvin J. Levy. "Fluorescence from the 1^1B_u state of *trans,trans*-1,3,5,7-octatetraene in a free jet". In: *The Journal of Chemical Physics* 81.4 (Aug. 1984), pp. 1592–1597. ISSN: 0021-9606. DOI: 10.1063/1.447888.

[10] Kevin L. D'Amico, Christopher Manos, and Ronald L. Christensen. "Electronic energy levels in a homologous series of unsubstituted linear polyenes". In: *Journal of the American Chemical Society* 102 (6 Mar. 1980), pp. 1777–1782. ISSN: 0002-7863. DOI: 10.1021/ja00526a003. URL: https://pubs.acs.org/doi/abs/10.1021/ja00526a003.

[11] Julio C. V. Chagas et al. "Low-lying excited states of linear all-*trans* polyenes: the σ–π electron correlation and the description of ionic states". In: *Physical Chemistry Chemical Physics* 27.15 (2025), pp. 7916–7928. ISSN: 1463-9076. DOI: 10.1039/D5CP00339C. URL: https://xlink.rsc.org/?DOI=D5CP00339C.

[12] Mario Barbatti, J. A. Adélia Aquino, and Hans Lischka. "A Multireference Configuration Interaction Investigation of the Excited-State Energy Surfaces of Fluoroethylene (C2H3F)". In: *J. Phys. Chem. A* 109 (23 2005). DOI: 10.1021/jp050834. URL: https://doi.org/10.1021/jp050834+.
